# An examination of positive selection and changing effective population size in Angus and Holstein cattle populations (*Bos taurus*) using a high density SNP genotyping platform and the contribution of ancient polymorphism to genomic diversity in Domestic cattle

**DOI:** 10.1186/1471-2164-10-181

**Published:** 2009-04-24

**Authors:** Sean MacEachern, Ben Hayes, John McEwan, Mike Goddard

**Affiliations:** 1 Primary Industries Research Victoria, Animal Genetics and Genomics, Attwood VIC 3049, Australia; 2 Latrobe University, Department of Genetics, Bundoora VIC 3086, Australia; 3 Animal Genomics, AgResearch, Invermay, Private Bag 50034, Mosgiel, New Zealand; 4 Melbourne University, School of Agriculture and Food Systems, Melbourne, VIC 3000, Australia; 5 Avian Disease and Oncology Laboratory 3606 E Mt. Hope Rd, East Lansing, MI 48823, USA

## Abstract

**Background:**

Identifying recent positive selection signatures in domesticated animals could provide information on genome response to strong directional selection from domestication and artificial selection. With the completion of the cattle genome, private companies are now providing large numbers of polymorphic markers for probing variation in domestic cattle (*Bos taurus*). We analysed over 7,500 polymorphic single nucleotide polymorphisms (SNP) in beef (Angus) and dairy (Holstein) cattle and outgroup species Bison, Yak and Banteng in an indirect test of inbreeding and positive selection in Domestic cattle.

**Results:**

Outgroup species: Bison, Yak and Banteng, were genotyped with high levels of success (90%) and used to determine ancestral and derived allele states in domestic cattle. Frequency spectrums of the derived alleles in Angus and Holstein were examined using Fay and Wu's H test. Significant divergences from the predicted frequency spectrums expected under neutrality were identified. This appeared to be the result of combined influences of positive selection, inbreeding and ascertainment bias for moderately frequent SNP. Approximately 10% of all polymorphisms identified as segregating in *B. taurus *were also segregating in Bison, Yak or Banteng; highlighting a large number of polymorphisms that are ancient in origin.

**Conclusion:**

These results suggest that a large effective population size (N_e_) of approximately 90,000 or more existed in *B. taurus *since they shared a common ancestor with Bison, Yak and Banteng ~1–2 million years ago (MYA). More recently N_e _decreased sharply probably associated with domestication. This may partially explain the paradox of high levels of polymorphism in Domestic cattle and the relatively small recent N_e _in this species. The period of inbreeding caused Fay and Wu's H statistic to depart from its expectation under neutrality mimicking the effect of selection. However, there was also evidence for selection, because high frequency derived alleles tended to cluster near each other on the genome.

## Background

Identifying positive genomic selection in domestic animals is a major challenge in contemporary agricultural research. To date only a small number of examples have successfully identified genomic regions subject to positive selection in domestic animals [1-10]. Increasing the understanding of positive selection and how it shapes genetic variation in domestic animals has the potential to provide powerful insights into the mechanisms involved in evolution, help target loci for selection and possibly highlight the genetic basis of phenotypic diversity for complex traits [5,11]. Domestic animals provide a unique opportunity to detect positive selection due to their extensive diversity amongst breeds, increasing availability of sequence data and large databases of polymorphisms that are accruing in domestic species like *Bos taurus*.

Data on polymorphisms can provide evidence of selection if the patterns in the data are incompatible with a neutral model [12]. For instance, the neutral model with constant effective population size predicts that most polymorphisms will have one common allele and one rare allele. More specifically, if p is the frequency of one of the two alleles chosen at random and f(p) is the distribution or spectrum of all polymorphisms where one allele has frequency p, then f(p) = k/(p(1-p)) where k is a constant. Tajima's D statistic [13] measures the extent to which real data differs from this theoretical expectation. Tajima [13] suggests that changes in the frequency spectrum of neutral polymorphic alleles can be used to detect a hitchhiking effect due to the spread of linked advantageous mutations. Therefore, high values of D indicate that common polymorphisms are more frequent than expected from the neutral theory and this is a result of genetic hitchhiking. However, polymorphisms are discovered by methods that tend to find common variants and this ascertainment bias can also generate an excess of polymorphisms with intermediate allele frequency.

The test for departure from expectation can be made more powerful if it is possible to distinguish the ancestral allele from the derived or mutant allele at each locus. If p is the frequency of the derived allele, then the distribution of all derived alleles is f(p) = k/p. Fay and Wu measure departure from this expectation with their H statistic [14]. If derived alleles are found at high frequency more often than expected, then H will be positive. They suggest that selection causes a positive H statistic, because selection sometimes drives the derived allele to high frequency. This can occur if the polymorphisms observed are subject to selection themselves, but can also occur at neutral loci as a result of hitchhiking caused by selection acting on linked loci. This makes H a very useful test for selection because most polymorphisms are discovered randomly and few of them are likely to be directly subject to selection.

Unfortunately, both D and H can depart from expectation for reasons other than selection [13,15-17]. The way in which polymorphisms are discovered usually means that low frequency polymorphisms are less likely to be discovered than one with alleles at intermediate frequency. D and H are also affected by changing effective population size (N_e_). If N_e _declines, polymorphisms with one rare allele become less frequent and the frequency spectrum becomes flatter. In this way a decline in N_e _(i.e. inbreeding) can mimic selection [16-18]. Therefore, detecting unambiguous examples of positive selection has been difficult due the difficulty of many methods to differentiate between positive selection and demographic history. This is of particular concern in domestic species where SNP discovery typically involves some ascertainment bias and demographic fluctuations coupled with strong directional (artificial) selection, which have played important roles in the formation of domestic breeds [19].

The problem of ascertainment bias will result in an observed allele frequency spectrum that is more flat than that predicted by theory. However, it is possible to construct a test that is not affected by this ascertainment bias if derived and ancestral alleles can be distinguished. Since f(p) = k/p for derived alleles with frequency p [14], the frequency spectrum for all ancestral alleles with frequency 1-p is f(1-p). The spectrum for all alleles with derived or ancestral allele frequencies p or 1-p is then f(p) + f(1-p), which is equal to f(p(1-p)), see above. So neutrality predicts that the proportion of these alleles where the ancestral allele is p is f(1-p)/[f(p)+f(1-p)], which is equal to p. Assuming that the polymorphism discovery method cannot distinguish ancestral and derived alleles, this expectation for different p intervals is not affected by the ascertainment bias. It has only been tested for p from 0 to 0.5, since the value of any f(1-p)/[f(p)+f(1-p)] is 1-(value at 1-p). Also, because selection does not typically affect all parts of the genome equally, selection and demographic phenomena can be compared. For instance, a selected allele can drag derived alleles that are closely linked to high frequencies by hitchhiking. Therefore, selection should cause an autocorrelation of high frequency derived alleles between one locus and the next on the chromosome. To test if the observed autocorrelation could be due to inbreeding, we have used a simulation study to demonstrate the effect of inbreeding in the absence of selection and compared the results with those found in real data.

Recently it has become possible to assay large numbers of polymorphisms in cattle and this offers a new source of data with which to detect evidence of selection. In this paper we use data from two breeds of cattle (Angus and Holstein) each genotyped for over 7,500 SNPs using the Parallele/Affymetrix platform. By also genotyping these SNPs on 3 species related to *Bos taurus *(Bison, Yak and Banteng) we have been able to distinguish the derived and ancestral allele at each locus and use this information to test for deviations from neutrality.

The comparison between the allele frequencies in the Angus and Holstein breeds might also contain evidence of selection since they have been selected for different traits. However, their allele frequencies also differ due to genetic drift caused by finite population size or inbreeding. The difference in allele frequencies can be quantified by the statistic Fst. Inbreeding should affect all loci equally and genetic drift should affect loci randomly and not show any linkage disequilibrium between adjacent loci, but we hypothesise that selection will drive linked derived alleles to high frequency in one breed but not the other. Therefore selection should cause higher values of Fst among loci where the derived allele is common than when the ancestral allele is common. We examine how Fst between Angus and Holstein changes with allele frequency and compare the result to that obtained with the simulated data.

## Results

### Amplification of *B. taurus *designed markers in wild relatives

Approximately 383 Holstein, 379 Angus and one of the outgroup species each (Banteng, Yak, and Bison) were genotyped for over 9,000 SNP. In total 7,611 amplified in both breeds of B. *taurus *and at least one of the outgroup species. Of these 6,718 and 7,215 SNP were found to be segregating in Angus and Holstein, respectively. The remaining assays were fixed for the derived or ancestral alleles (table 1).

**Table 1:** Summary of successful genotypes for loci amplified in Angus, Holstein and at least one wild species

	Segregating	Fixed ancestral	Fixed derived	Total
Angus	6,718	809	84	7,746
Holstein	7,215	373	23	7,746

Table 2 summarises the proportion of successful assays in the outgroup species sampled from 9,323 SNP genotyped in Angus. On average very high rates of successful assays were found in the majority of wild species. Similar results have been found for the successful amplification of SNP markers as for successful amplification of exons from primers designed from the flanking 5- and 3- prime regions of each exon [20]. From the 8,677 loci that amplified in the wild species 7,611 could be used to identify the ancestral allele, while 931 were determined to be polymorphic or segregating in the ancestral species.

**Table 2:** Proportion and numbers of alleles showing successful genotypes in individual wild relatives and overall total where at least one animal was successfully genotyped from the 9,323 SNP genotyped in Angus

	Bison	Yak	Banteng	Water Buffalo	≥ 1 species
Proportion	0.924	0.923	0.923	0.0	0.931
Genotypes	8,615	8,608	8,607	0	8,677

### Fay and Wu's H test

In table 3 a summary of Fay and Wu's H statistic is presented for a survey of 7, 611 SNP in Holstein and Angus cattle. In both Angus and Holstein populations H values are considerably larger than 0 (p < 0.001, one sided). A comparison of H values between Holstein and Angus identified a significantly larger value for H in Angus cattle (p < 0.001). This indicates that derived alleles occur at a higher frequency than expected under the neutral model, especially in Angus.

**Table 3:** Summary of H statistics from a survey of over 7,746 SNP in Holstein and Angus cattle

	Sample size	θ_H_	θπ	H	s.e.(H)	p(H)
Angus	7,746	0.448	0.274	0.174	0.0065	p < 0.001
Holstein	7,746	0.435	0.316	0.119	0.0061	p < 0.001

*s.e.(H) is the standard error of H and p(H) is the probability that θ_H _and θπ are equal

#### Frequency spectrum of derived and ancient alleles

**Figure 1:**
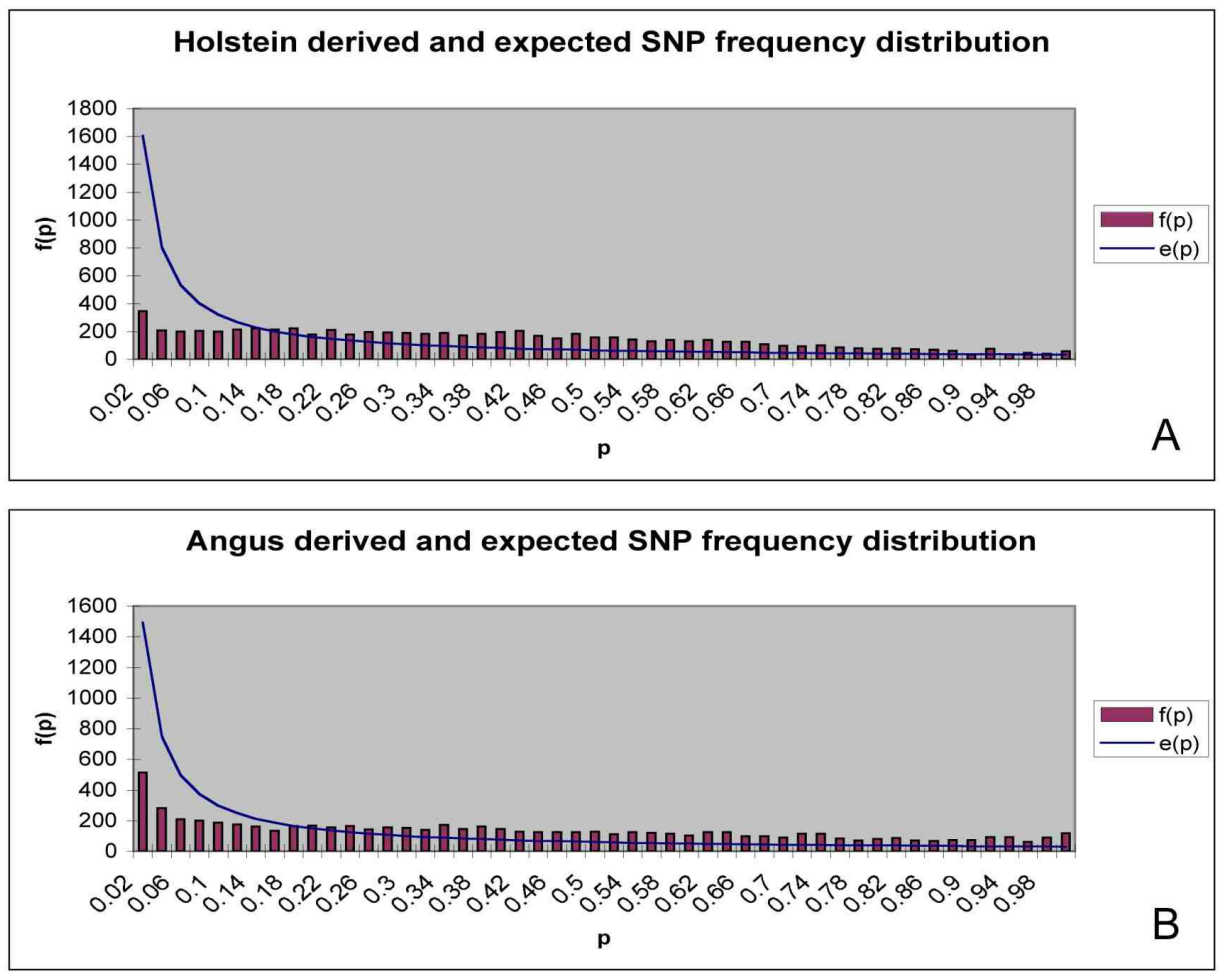
**Frequency spectrum of the derived alleles for Holstein (A) and Angus (B), plotted as f(p) against p and the expected distribution under neutrality k/p, where p = the frequency of the derived allele**.

The frequency spectrum of derived variants f(p) alongside the expected frequency spectrum e(p) under neutrality, which is calculated as k/p where k = the value calculated from the sum of all f(p) so the theoretical and actual curves match as close as possible for Holstein and Angus cattle, is presented in figure 1A, B. In general both breeds show a flat distribution for the spectrum of derived alleles. Both Holstein and Angus show a deficiency of derived alleles at low frequency, especially in Holstein. However, at high frequencies there may be an increase in the abundance of derived alleles, especially in Angus cattle.

**Figure 2:**
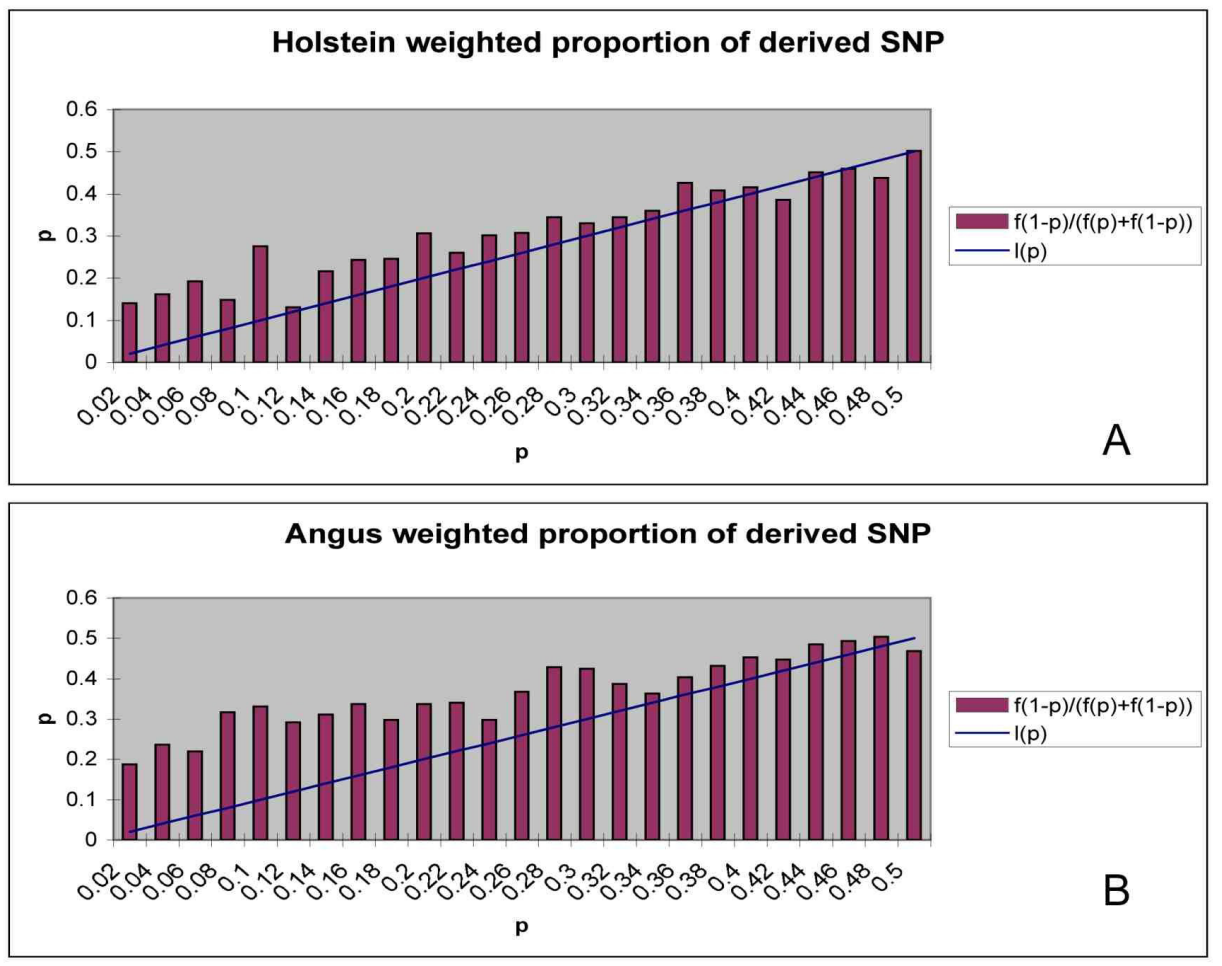
**frequency of derived alleles corrected for ascertainment bias in Holstein (A) and Angus (B) and expected value under neutrality, l(p)**.

To avoid ascertainment bias, we have plotted f(1-p)/[f(p)+f(1-p)] against its expectation under the neutral model in figures 2A and 2B. Under a neutral model with constant N_e_, the expectation of this statistic is simply p. The figures show that the derived alleles are more common than expected. For instance, in figure 2A when the minor allele frequency is 0.1, we expect that in 0.1 of the loci the common allele is the derived allele, but we observe this in 0.27 of the loci. In Holstein and especially Angus, derived alleles are more common than expected.

### Genomic distribution of high frequency derived polymorphisms

The frequency spectrum of derived alleles appears to follow a pattern contrary to that expected under neutrality, as there are too many derived mutations with relatively high frequency. If these mutations are clustered in certain areas of the genome, this may be evidence of positive selection driving these alleles to high frequencies. Alternatively if they are distributed randomly throughout the genome, this may be a result of inbreeding randomly increasing the frequency of some derived (new) mutations.

The distribution across the genome of high frequency derived alleles in Angus and Holstein was compared to that in two simulated populations devoid of positive selection to determine if the pattern observed in Angus and Holstein could be due to changing N_e _or whether it is indicative of selection. Table 4 summarises the results of an analysis using autocorrelation between adjacent and derived polymorphisms per chromosome for both *B. taurus *and simulated populations. Positive correlations were detected in the *B. taurus *breeds, while the autocorrelation in the simulated data did not differ significantly from zero. A t-test indicated that on average the positive correlations found in Holstein and Angus were significantly larger than zero, while those in the simulated populations were not (α = 0.05, two sided). Thus, high frequency derived alleles seem to be clustered in the genome, especially in Holstein.

**Table 4:** Autocorrelation for derived allele frequency and the frequency of adjacent derived polymorphism for *B. taurus *and simulated populations

Chromosome	Angus	Holstein	Simulated 1	Simulated 2
1	0.054	0.004	0.301	0.17
2	0.141	0.16	-0.009	0.075
3	0.011	0.028	0.146	-0.146
4	-0.014	0.007	-0.069	-0.123
5	0.113	0.142	-0.003	0.007
6	0.073	0.036	-0.109	0.002
7	0.125	0.082	0.293	0.178
8	0.145	0.093	-0.063	0.055
9	-0.013	-0.005	0.086	-0.042
10	0.031	0.055	-0.211	-0.012
11	0.063	0.095	0.002	0.066
12	0.098	0.16	0.014	0.087
13	0.196	0.244	0.069	0.143
14	0.068	0.155	-0.081	-0.057
15	0.047	0.043	0.089	0.102
16	-0.017	0.024	-0.073	-0.023
17	-0.042	-0.083	0.002	0.014
18	0.055	0.077	-0.072	-0.271
19	0.007	-0.048	-0.046	-0.136
20	0.034	0.034	0.063	0.015
21	-0.037	-0.023	0.078	-0.132
22	0.029	0.026	-0.141	0.042
23	0.11	-0.035	-0.021	-0.161
24	-0.07	0.017	-0.055	-0.024
25	0.127	0.211	0.109	-0.12
26	0.082	0.252	-0.042	0.104
27	0.017	0.134	-0.155	0.049
28	-0.035	-0.1	0.035	-0.117
29	0.175	0.096	0.149	0.02
X	0.056	0.085	-	-
Average (s.e)	0.054 (0.012)	0.066 (0.016)	0.01 (0.022)	-0.008 (0.02)
Pvalue	p < 0.001	p < 0.001	p > 0.05	p > 0.05

*X chromosome not modelled in simulated populations

In order to visually summarise the data presented in table 4 we plotted the derived allele frequency against genomic position over the entire genome, we also contrasted this with Fst at the same positions. Due to the volume of information, these plots have been included as supplementary figures 1–30 A and B for all Holstein and Angus chromosomes (see Additional files 1, 2, 3, 4, 5, 6, 7, 8, 9, 10, 11, 12, 13, 14, 15, 16, 17, 18, 19, 20, 21, 22, 23, 24, 25, 26, 27, 28, 29 and 30). In general if we just examine the derived allele frequency some grouping of derived alleles at high frequencies can be found throughout the genome. The largest autocorrelations were found on chromosomes 2 and 13 in Holstein and Angus (Table 4, Additional files 2 and 13). This could be due to positive selection independently increasing the frequency of derived polymorphisms in both breeds, or more likely it could be due to selection acting in the common ancestor of Holstein and Angus. For chromosomes 25, 26 and 29 high autocorrelations in one breed and not the other where identified, which may be evidence of breed specific selection (Table 4, Additional files 25, 26 and 29). Also, lack of any significant correlation in the simulated populations in table 4 suggests that inbreeding cannot solely create this clustering of high frequency derived alleles.

### Phylogenetic analysis and the frequency of derived alleles in Holstein and Angus

We used a neutral phylogenetic tree to overlay the average frequency of derived alleles between Angus and Holstein cattle (data not shown). At the 7,611 polymorphic sites analysed, the Holstein breed had a higher average derived allele frequency (0.362) when compared to Angus (0.359), which is similar to the finding we observed in Table 4. However, the difference between breeds was not significant.

### Average Fst and derived allele distributions

**Figure 3:**
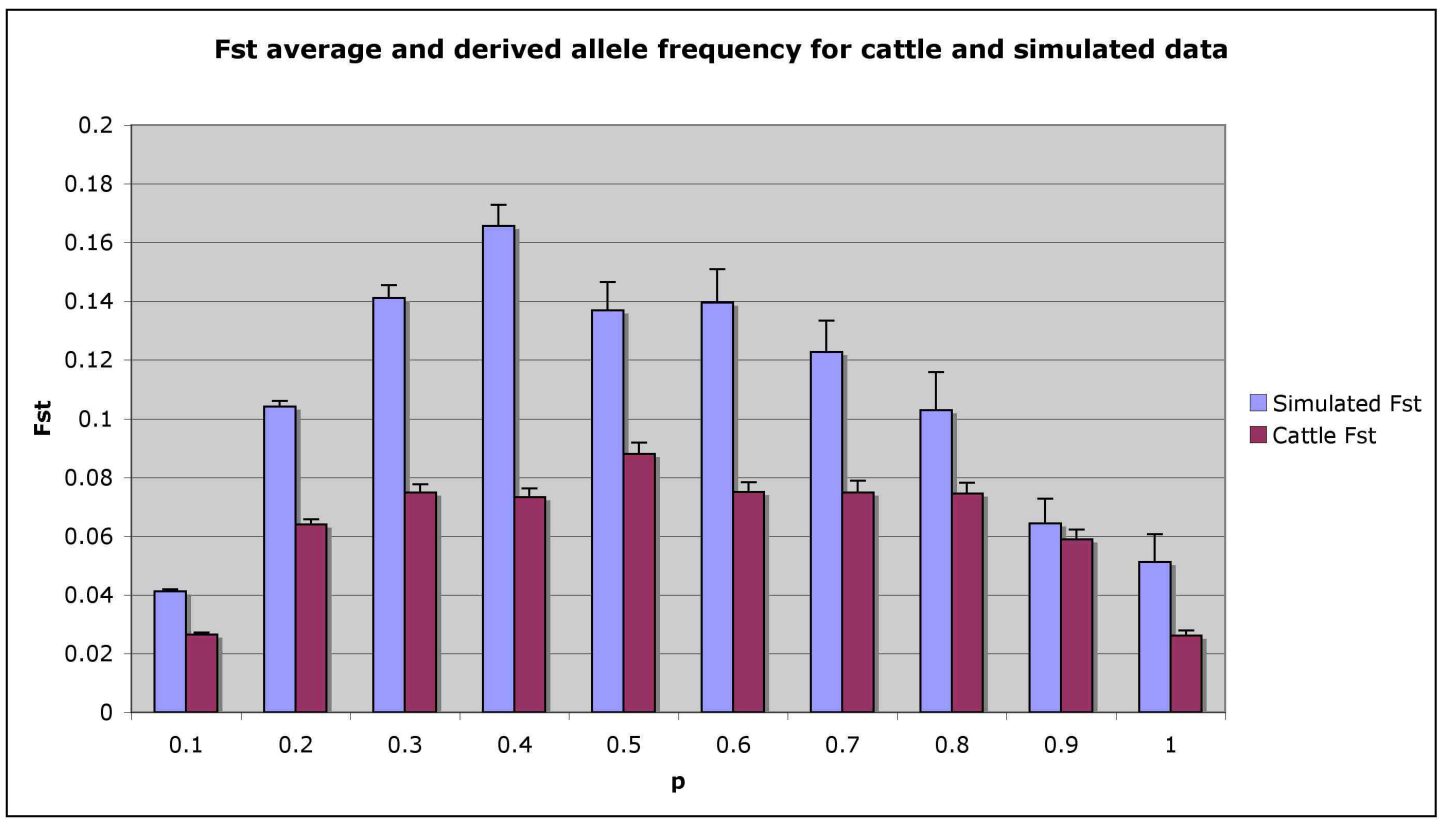
**Histogram of the Fst value between the simulated populations and Holstein-Angus cattle for the average derived allele frequency (p), error bars represent the standard error estimates**.

Fst between Holstein and Angus was 0.07 and between the two simulated populations was 0.09. In figure 3 Fst is calculated for specific ranges of derived allele frequencies (0–1.0). For both the simulated and real populations, Fst is highest when the average allele frequency is 0.4 or 0.5, which is a feature of the formula for Fst. We hypothesised that selection would create higher Fst values when the derived allele was frequent than when it was rare, but there is no evidence for such a trend when the real populations are compared to the simulated populations. In general higher Fst detected between simulated populations than between *B. taurus *breeds probably reflects the simulation and an over estimate of the effect of inbreeding in the theoretical populations. However, the differences between the frequency spectrums are small.

**Figure 4:**
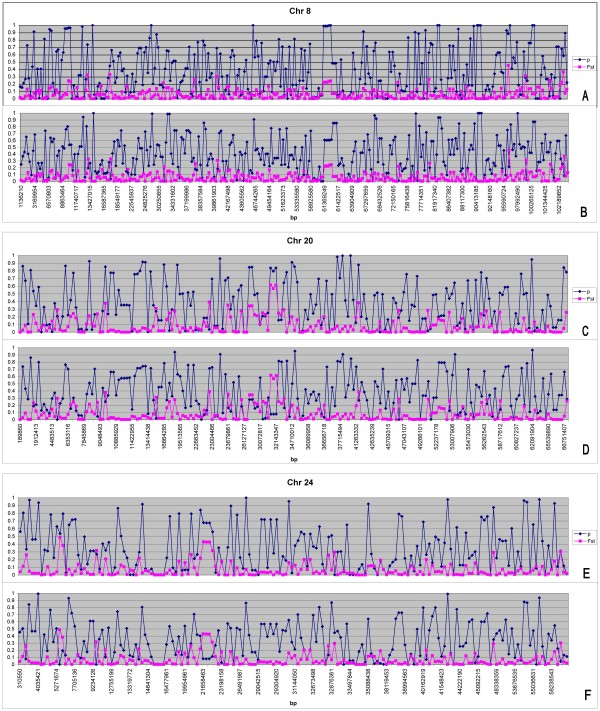
**A-F Chromosome plots of Fst and derived allele frequency (p) in relation to genomic position (bp) for Angus (A, C and E) and Holstein (B, D and F)**.

The distribution of derived allele frequencies and Fst are quite different analyses of the polymorphism data. Given that a number of chromosomes in table 4 have shown different degrees of clustering for high frequency derived alleles. We plotted the genomic distribution of Fst and the derived allele frequency (p) per chromosome to see if any genomic regions were outliers for both (see Additional files 1, 2, 3, 4, 5, 6, 7, 8, 9, 10, 11, 12, 13, 14, 15, 16, 17, 18, 19, 20, 21, 22, 23, 24, 25, 26, 27, 28, 29, 30). Overall the majority of chromosomes in additional files 1, 2, 3, 4, 5, 6, 7, 8, 9, 10, 11, 12, 13, 14, 15, 16, 17, 18, 19, 20, 21, 22, 23, 24, 25, 26, 27, 28, 29, 30 show genomic regions where there are outliers for high Fst and derived allele frequency. In particular, one region on chromosomes 8, 20 and 24 (Figure 4A–F) stands out in Angus between base pairs 61,300,000–62,500,000, 3,210,000–3,400,000 and 21,600,000–22,200,000, respectively. At these regions of the genome show derived alleles in Angus that have been driven to a frequency of 0.7–1.0, while in Holstein they range from 0.1–0.6. This difference in derived allele frequencies has been responsible for an inflated Fst value (0.2–0.6), when compared to the average between breeds (0.07). An examination of these regions identifies no particular candidates of positive selection, except for some unknown genes and FGF1 (fibroblast growth factor1) on chromosome 20. However all of these chromosomal regions show evidence of QTL affecting body composition (Bta 8, Bta 24) and carcass yield (Bta 20) [21,22]. QTL resolution remains coarse for these regions, as the cattle genome becomes more completely annotated these regions may identify useful targets of selection.

### Frequency spectrum of ancient polymorphisms in *B. taurus*

In total the 8,677 SNP markers in Angus that successfully genotyped in at least one of the wild species were examined for evidence of ancestral polymorphisms. Of these, we identified 931 (10.7%) that appear to be ancestral in origin, in that the three groups were not fixed for a single allele (see Methods).

**Figure 5:**
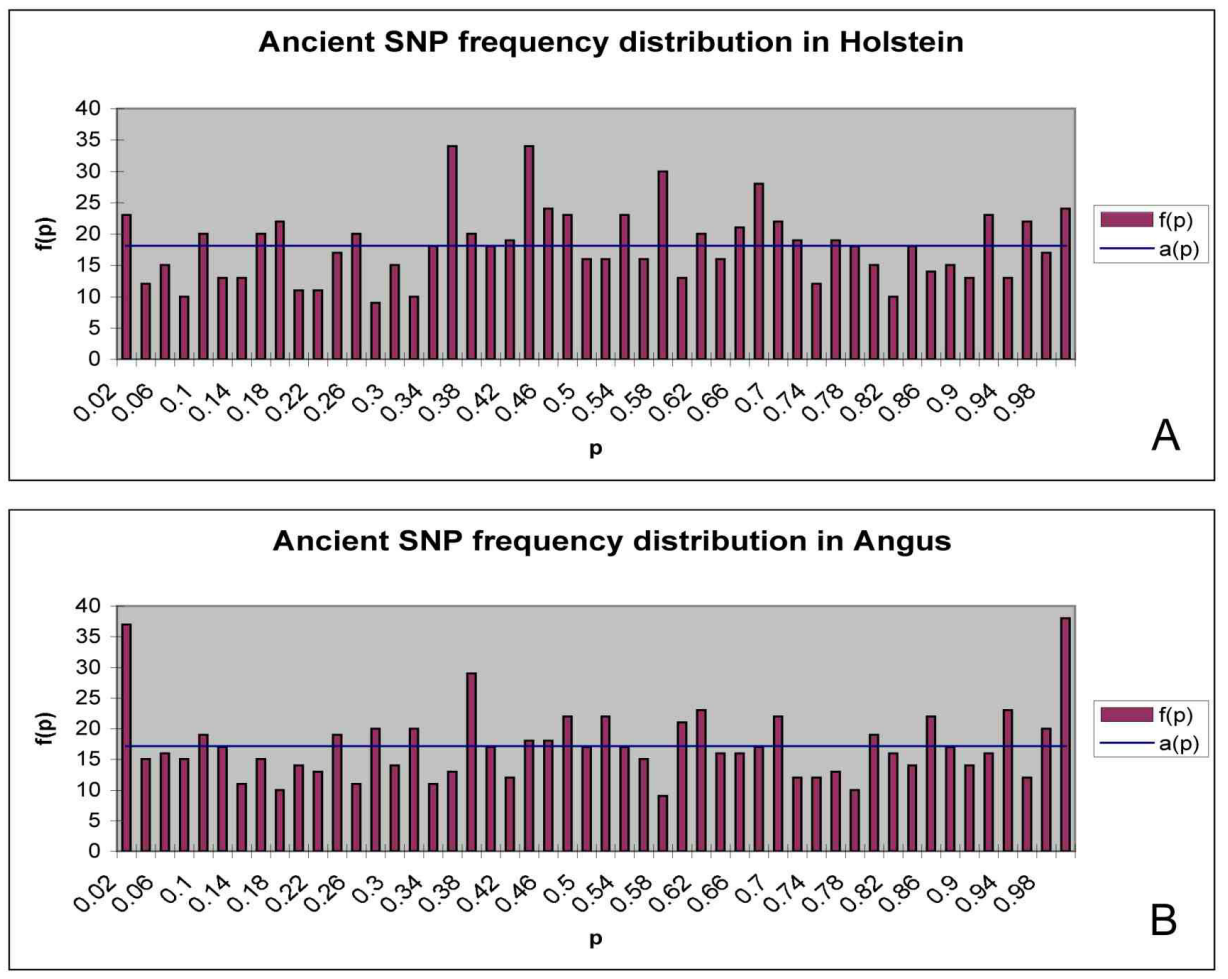
**Frequency spectrum of rare and common alleles for Holstein (A) and Angus (B) as determined by ancestral polymorphisms segregating in wild relatives, plotted as f(p) against p and the expected distribution under neutrality e(p)**.

In figures 5A and 5B the frequency spectrum of ancestral polymorphisms along with the expected distribution in Holstein and Angus are presented, respectively. The derived and ancestral alleles could not be determined for these polymorphisms, because the SNPs are segregating in the wild relatives. Therefore an allele was chosen at random and its allele frequency was used in figure 5. In both breeds the frequency spectrum of ancestral polymorphisms is generally flat. However, there is an excess of rare (< 0.02) and most common (> 0.98) classes of alleles, some of which may be typing errors.

#### Ancestral polymorphism and effective population size

The 931 ancestral polymorphisms found in *B. taurus *suggest that despite any recent bottlenecks in *B. taurus *that occurred during the domestication process, very large populations must have been maintained in the ancestral Auroch (*B. taurus primigenius*) population prior to its domestication some 10,000 years ago [23-25]. Thus, 931/8,677 = 0.11 of SNP in *B. taurus *have remained polymorphic for over ~2 million years.

Hence, if the fixation of neutral alleles is typically governed by chance, the probability of fixation per generation after equilibrium is reached is

(1)

and the probability of no fixation over t generations is

(2)

For the purpose of estimating the effective population size of the ancestral species it was assumed that the number of polymorphisms was similar between ancestral and contemporary populations. The divergence times estimated by MacEachern et al [20] were used to calculate the average divergence time for Bison, Yak and Banteng, which was approximately 2.1 MYA. If the average generation time for these animals is 5 years, t should be roughly 4.2 × 10^5 ^generations. Thus, for equation 2, setting e^-t/2Ne ^= 0.11, gives an effective population size of ~90,000 animals, and of course the actual number of animals would have been some degree larger than this. It is important to note that this estimate is affected by our estimate of generation time, which may have been greater than 5 years. However, the rate of fixation in domestic cattle most likely increased with decreasing population size and therefore our estimate should be a good approximation.

### Ancestral alleles and phylogenetic relationships in closely related bovids

From the 8,677 SNPs assayed in at least one wild relative, 931 loci could not have an ancestral allele determined as both alleles were found to be present in at least one of the three closely related species. From these 931 SNPs the number of cases where both *B. taurus *alleles are detected in one of the three related species is summarised in table 5. MacEachern et al. [20] concluded that the Yak appeared to share more alleles with *B. taurus *than Bison, although this difference was not significant using a t-test (α = 0.05, one sided). In the present study Banteng was found to show roughly double the number of mutations in common with *B. taurus *than Bison or Yak and was significantly more similar to *B. taurus *than the other two species (α = 0.05, one sided). However, our Banteng samples were sourced from the Taronga Western Plains Zoo in Australia and there was no guarantee that this animal had no *B. taurus *ancestors. The possibility that the Banteng has been crossbred may potentially affect the estimates of N_e_, as some of the 931 alleles originally thought to be ancestral polymorphisms could be the result of recent introgression. However, this should only affect a small percentage of the 931 SNPs and therefore not have a large impact on the estimate of N_e_. Although without 100% surety of the genetic purity of our wild relatives some caution may be needed for the interpretation of our findings

**Table 5:** Examination of Bison, Yak and Banteng where both *B. taurus *alleles are detected

Bison	Yak	Banteng	Count
0	2	2	63
1	0	0	15
1	2	2	21
2	0	0	65

Bison anomaly grand total:			164

0	2	0	67
0	1	0	24
2	0	2	74
2	1	2	25

Yak anomaly grand total:			190

0	0	2	141
0	0	1	46
2	2	0	120
2	2	1	42

Banteng anomaly grand total:			349

0 = homozygote allele A, 2 = homozygote allele B, 1 = A/B heterozygote

#### Heterozygosity

As only one animal was sampled for each wild species their heterozygosity was estimated as the proportion of heterozygotes from the total number of SNPs that successfully amplified in each animal. Table 6 summarises the number of heterozygotes detected from the number of successful assays and the average heterozygosity for each sample. The highest heterozygosity was found in Banteng and the lowest was found in Bison.

**Table 6:** Number of heterozygous markers, the proportion they contribute to aberrant SNPs and the average heterozygosity calculated from the total of successfully amplified markers

Species	Heterozygotes	Proportion of aberrant SNPs	Total SNP	Average heterozygosity
Bison	87	0.09	7,698	0.0113
Yak	96	0.1	7,635	0.0126
Banteng	144	0.15	7,681	0.0188

## Discussion

### Genotyping and wild specimens

We have found that a high proportion (~0.9) of SNP markers, designed from the *B. taurus *genome, were successfully assayed in a number of wild relatives from the Bovina subtribe, which diverged from *B. taurus *between 1–2 MYA, while no successful genotypes were recorded from the Bubalina subtribe, which diverged some < 9 MYA. It is unclear whether the lack of amplification in the Bubalina was the result of low selective constraint over SNP, or if there was a problem with DNA. However, a recent study by Gautier et al. [19] identified a high rate of successful SNP assays in goat (*Capra hircus*), which diverged at least 15 MYA from cattle. Thus members of the Bovina subtribe should be suitable outgroups for SNP genotyping studies in *B. taurus*.

### Genome wide polymorphism scans reveal a deviation from neutrality in two breeds of *B. taurus*

Researchers utilising the bovine genome have uncovered large number of SNPs, which has presented researchers the opportunity to examine patterns of DNA sequence variation for indirect evidence of positive selection by cataloguing levels of genetic hitchhiking. To this end, we analysed over 7,500 SNP spaced throughout the genome of Holstein and Angus cattle (*B. taurus*). We identified significantly large deviations from neutrality in each *B. taurus *population using the H statistic developed by Fay and Wu [14], which may result from either recent positive selection, ascertainment bias or inbreeding.

Scans examining the frequency distribution of polymorphisms for deviations from neutral expectations often run into difficulty when trying to differentiate between the effects of positive selection and demographics. This is because the null hypotheses used to test for significance unrealistically assumes that the demographic history of the sample population was a random mating population with an unchanged N_e _[16,26]. Ascertainment bias for common alleles can also affect scans for positive selection from polymorphism data, which may mimic the results expected for positive selection or those produced from demographic processes such as inbreeding. The ~10,000 SNPs that were provided as part of the genotyping platform by Parallele Bio Sciences were discovered using the cattle genome project, based on sequences from only one or a few animals (information regarding their discovery is at ). The small numbers of animals sampled during SNP discovery would suggest that there is some ascertainment bias. Hence, inferences of positive selection from tests that come directly from allele frequency distributions should be made with caution.

By examining several thousand loci it was possible to distinguish between the effects of population structure, positive selection and ascertainment bias on the frequency spectrum of ancestral and derived alleles. As indicated by the significant H test, the results detected an excess of high frequency derived alleles in Holstein and Angus populations. The frequency spectrums in figure 1 are generally too flat when compared to the distributions expected under neutrality. The frequency distribution highlights a number of discrepancies at medium frequencies (0.4–0.6) for Angus and Holstein, which would be expected if there was ascertainment bias towards common polymorphisms. However, a flat distribution was also found in figure 2 and this plot removes any effects from ascertainment bias on the frequency distribution as the plot examines the frequency of the derived allele compared to the ancestral allele, using the ratio f(1-p)/[f(p)+f(1-p)] and information regarding the derived and ancestral alleles are irrelevant during the SNP discovery. Hence, the flat distribution witnessed in figure 2 indicates that derived alleles occur at high frequency more often than expected and that this was not due to any ascertainment bias. This new metric may be of use to researchers trying to identify selective sweeps to datasets that are influenced by ascertainment bias.

Reduced N_e _or inbreeding could, however, cause the distribution observed in figure 2 because it leads to a random dispersal of allele frequencies. We used a transition matrix method to calculate the amount of inbreeding necessary to generate an allele frequency spectrum that matched that observed. This was done by starting with the allele frequency spectrum expected under the neutral model (ie f(p) = k/p) and using a transition matrix which calculated the spectrum one generation later assuming a population of effective size N_e _and no mutation. The matrix multiplication was repeated multiple times until the spectrum matches the observed spectrum. We found that to replicate a frequency distribution similar to that displayed in figure 1 would require enough generations to reach an inbreeding coefficient of 0.5.

An example of the effect of genetic drift is shown by the frequency spectrum of ancient polymorphisms presented in figure 2, which shows a very flat distribution. This is expected for very old polymorphisms. It is unlikely that these polymorphisms have all been maintained due to overdominance and so we assume that they have been maintained in the historically large populations that once existed in bovids. Slight peaks are witnessed at the extremes of the distribution < 0.02 and > 0.98. Because all fixed alleles with frequencies of 0 and 1.0 were removed from the analysis, these may represent typing errors or be evidence of alleles that have been positively selected towards fixation.

#### Genomic distribution of high frequency derived alleles

The large number of high frequency derived alleles found in Angus and Holstein populations are unexpected under neutrality with constant N_e _[14]. To distinguish between the effects of positive selection and inbreeding on the frequency spectrum of derived mutants in *B. taurus *we examined the tendency of derived alleles to cluster together in the genomes of Angus and Holstein populations using the autocorrelation between frequencies of derived alleles. A positive autocorrelation for derived allele frequencies between neighbouring loci indicated that there is an association between high frequency derived alleles in the genomes of both cattle breeds. This is consistent with positive selection and not changes in population size or ascertainment bias. A simulated population that had been inbred to similar levels as contemporary populations, without the influence of positive selection, failed to show a similar autocorrelation between high frequency derived alleles. Therefore, this suggests that hitchhiking events are common throughout the genomes of both breeds of *B. taurus *and this is consistent with positive selection for some loci. If this is the case, it appears to have influenced the Holstein genome more than the Angus genome. There is a possibility that our findings are the result of sampling error. However, as this study is based on a fairly large sample size in both breeds (n > 300) we believe the findings are indicative of stronger artificial selection in the Holstein breed.

#### Fst distribution and inbreeding and selection in B. taurus

If different selection pressures operated in Holstein and Angus, then different derived alleles might be driven to high frequency in the two breeds. This would cause Fst between the breeds to be higher when the frequency of the derived allele was high. We estimated Fst per locus and plotted their average values against the average derived allele frequency in *B. taurus *and in the simulated populations (Figure 3). Initial examinations of the frequency distribution of Fst between *B. taurus *breeds do not appear to find any overwhelming signatures of positive selection as the distribution of Fst is fairly symmetrical with respect to allele frequency. Nor has examining Fst plots for the simulated and observed populations identified any convincing differences. However, plots of derived allele frequency overlayed with Fst identified regions on chromosomes 8, 20 and 24 in Angus where large regions have had the derived alleles driven to near fixation generating higher Fst values for these regions than the overall average (Figure 4A–F). A quick examination of the genes underlying these regions has not identified any remarkable candidates for positive selection, perhaps except for FGF1 in beef cattle, but these regions are associated with QTL identified for body composition and carcass yield [21,22]. Hence, our results may be of future interest for identifying signatures of recent positive artificial selection between the two breeds or as additional evidence for any polymorphisms that show associations with beef or milk traits.

### Ancestral polymorphisms

A recurring theme that appears when examining polymorphisms in *B. taurus *is the high proportion of polymorphisms that appear to be segregating in wild species. In the phylogenetic analysis presented by MacEachern et al. [20] a surprisingly large proportion (8.7%) of nucleotide substitutions between species did not follow a simple tree implying that these sites were polymorphic in an ancestral species that had subsequently undergone lineage sorting in the extant bovids. These ancestral polymorphisms were found from sequencing a number of *B. taurus *breeds and a subset of wild relatives from the Bovinae subfamily without any knowledge about whether they were still segregating in *B. taurus*. In contrast, work completed in the current study focuses on nucleotides that have been identified to be segregating in *B. taurus*. However, we have also found a large proportion of *B. taurus *polymorphisms (10.7%) to be segregating in the wild relatives. Despite the difference in the method by which they were detected, the possible explanations for them are similar. That is, they must have arisen due to a double mutation, introgressive hybridisation or alternatively they are due to the presence of ancestral polymorphisms that are still segregating in *B. taurus*. These loci may in fact still be segregating in the wild relatives, even if they are not heterozygous in the wild species sampled. Alternatively, they have undergone lineage sorting and hence they appear to be alleles with 'abnormal' inheritance, as described in MacEachern et al. [20,27]. Therefore, approximately 10% of all polymorphisms in the Bovinae are likely due to ancestral polymorphisms in the common ancestor of cattle and Bison/Yak/Banteng.

MacEachern et al. [20,27] have suggested that the majority of ancestral polymorphisms are neutral and thus their frequency is governed by random genetic drift, suggesting they have persisted in the extant members of the Bovinae due to chance. In the current paper the frequency spectrum of ancient polymorphisms presented in figure 4 is largely flat for both populations of *B. taurus*, especially if the extremes of the distribution are ignored because they are most likely to represent typing errors. Thus the flat distributions in figure 4 suggest that the ancient polymorphisms are largely neutral. Hence, these sites should be useful for determining the effective population size in ancestral species, which we estimated to be approximately 90,000. This estimate of N_e _can be quickly compared with the heterozygosity expected in contemporary populations of *B. taurus *as the heterozygosity expected is

(3)

where μ = the mutation rate per generation. If the mammalian mutation rate is 2.2 × 10^-9 ^per base per year as estimated by [28] this implies 1.1 × 10^-8 ^mutations per base per generation if the generation length is 5 years. Then equation 3 gives He = 0.0035. MacEachern et al [25] sequenced eight Holstein animals and the polymorphism rate in noncoding DNA was estimated as 0.00373. We have used a modification to equation 2 in Fay and Wu [14] to convert this to an estimate of heterozygosity within Holstein to compare this result with the estimated heterozygosity from equation 3. Hence, Holstein heterozygosity was estimated as

(4)

where *dI *is the polymorphism rate at noncoding sites estimated from MacEachern et al. [20] and n = the number of chromosomes sampled. This yields an estimate of heterozygosity in Holsteins equal to 0.0011, which is approximately 3 times less than what we expected from N_e _= 90,000 (He = 0.0035). This difference could largely be the result of the amount of inbreeding that has occurred in contemporary populations of *B. taurus *or by error in the estimate of μ.

#### Wild species heterozygosity and overall similarity to B. taurus

A number of the ancestral polymorphisms were examined for the proportion of loci that were heterozygous in the outgroup species and for their similarity to *B. taurus*. MacEachern et al. [20] identified a large number of genetic similarities between Yak and *B. taurus*. We have found that Yak shares slightly more alleles with *B. taurus *than does Bison, but the difference was not significant. Thus, there is only weak evidence that Yak is more closely related to *B. taurus *than is Bison. We have also found that Banteng shared a larger number of alleles with *B. taurus *than Yak or Bison, which is most likely a result of having a Banteng animal with questionable ancestry, which may result in a slight over estimation of the number of ancestral polymorphisms and hence of N_e_. If the Banteng sample contained *Bos taurus *genes, it would inflate the number of cases where Banteng was heterozygous for the Yak/Bison allele and the *B. taurus *allele. Table 5 shows that this occurred in only 88 cases out of 931 SNPs. Therefore, even if this hybridisation had occurred, it would not affect the conclusions greatly.

Examining the proportion of heterozygous loci in table 5, Yak appears to be more heterozygous than Bison, which may reflect past population bottlenecks in Bison [29,30]. Not surprisingly, Banteng was the most heterozygous of all animals, and this might be expected if the Banteng has a questionable background. Although we believe the Yak and Bison samples used are genetically pure, without 100% certainty about the ancestry of our samples, some caution may be needed with our interpretations that rely on this aspect.

## Conclusion

We have examined the frequency distribution of polymorphisms in milking and beef breeds of *B. taurus *using Fay and Wu's H as a test to identify genomic positive selection. Significant deviations from neutral expectations were identified, which appears to be a combined effect of positive selection, inbreeding and ascertainment bias for common polymorphisms. By distinguishing derived from ancestral alleles we were able to eliminate the effect of ascertainment bias from our test for selection using a new metric f(1-p)/[f(p)+f(1-p)] that is able to overcome many of the problems associated ascertainment bias when knowledge of the ancestral state is known. This metric could potentially be useful for a number of studies that rely on information from allele frequency distributions. The high frequency of derived alleles we have identified here could be caused either by selection or reduced N_e_. Reduction in N_e _appears to have occurred because the ancestral N_e _predicts a higher herterozygosity than observed. However, the tendency of high frequency, derived alleles to cluster in certain parts of the genome is evidence for positive selection because inbreeding alone does not cause this autocorrelation.

By including a number of wild relatives in the analysis the ancestral alleles were inferred. Surprisingly a high proportion of ancestral polymorphisms were identified suggesting that nearly 10% of all of the polymorphisms that are segregating in contemporary populations of *B. taurus *are ancient in origin and must predate the divergence of Bison, Yak, Banteng and the Domesticated cow. These ancestral polymorphisms were therefore used to estimate the ancestral population size of domesticated cattle over the last 2 million years, which must have been at least 90,000. This estimate is roughly 9 times greater than the estimate of the effective population size in humans, which has been estimated as 10,000 [31].

## Methods

### Sample Animals

Two separate breeds of *Bos taurus *and a number of wild relatives were analysed for genotypic polymorphisms using a high-throughput, high-density SNP genotyping platform. This platform is commercially available from Parallele Biosciences, which was acquired by Affymetrix . The original progenitors of the Angus and Holstein breeds are thought to be have existed for over two thousand years in Scotland and Germany/North Holland, respectively. However breed development did not occur until the early to mid 1800's [32]. The breed histories are very similar in that, during the past 50 years Angus and Holstein have experienced dramatic increases in selection pressure for beef and milk production, respectively and decreases in effective population size to approximately 100 individuals each [33].

Angus animals were selected from Trangie Agricultural Research Centre in NSW, Australia. All animals had information on sire and dam pedigree records, were born from 1993 to 2000 and had been selected for high or low post-weaning feed efficiency (FE), or were part of a control herd. Holstein animals were selected from a research project based at Genetics Australia in Victoria, Australia. All animals were bulls selected as semen donors for artificial selection, have information on pedigrees and have been selected for high and low estimated breeding values (EBVs) as determined by Australian Selection Index (ASI), which is an economic index of milk, fat and protein yield from bulls' daughters via progeny testing. Approximately equal numbers of the extreme highest and lowest FE and ASI animals were selected for SNP genotyping. A single Yak (*Bos grunniens*), North American Bison (*Bison bison*), Banteng (*Bos javanicus*) and Water buffalo (*Bubalus bubalis*) were chosen as outgroup species and genotyped in an effort to infer derived and ancestral alleles in both populations of *B. taurus*. Thus, there were 762 *B. taurus *animals and 4 wild relatives genotyped for a number of SNP markers, using the Parallele™ technology.

### Genotyping

For each of the 766 animals, DNA was extracted from blood or semen and DNA samples were diluted to 30 ng/ul. In Angus and the four wild species 9,323 SNPs, distributed across the bovine genome were genotyped at Parallele Bio Science Inc. There were slight differences in SNP platforms as a result of Parallele Bio Sciences being taken over by Affymetrix Inc., thus, a total of 9,919 SNP were genotyped in Holstein. Only the polymorphisms genotyped on both breeds were compared.

### Analysis

We used the Python programming language to parse data files and extract genotypes for all animals at each locus and calculate frequencies of derived and ancient alleles in Angus and Holstein populations.

#### Ancestral and derived alleles

Ancestral alleles were determined using outgroup species. For loci where only one allele was represented in the wild relatives, that allele was determined as ancestral. Loci where both alleles were represented among the outgroup species were considered ancient polymorphisms that must have arisen at least 2 MYA, before the separation of the Bison, Yak, Banteng and *B. taurus*.

#### Genomic position of Parallele polymorphisms

The genomic position of all Parallele SNPs were determined by comparing the flanking sequence to the Bovine genome (Btau_3.1) scaffolds using the BLAT algorithm [34]. Results are presented in the genome browser of the Interactive Bovine In Silico SNP (IBISS) database.

#### Computer simulations

A computer simulation was developed to determine the probability that the observed differences in allele frequencies between breeds were due to finite N_e _without selection. A diploid population, of N_e _= 50,000 was simulated with mutation and recombination until an equilibrium was reached. Then N_e _was reduced to 1,000, was simulated for 1,000 generations. In reality, estimated values of N_e _for early domestic *B. taurus *some 2,000 generations ago was closer to 1,500 [35]. However, for computational ease N = 1,000 and 1,000 generations was chosen. Each individual in the population consisted of 29 pairs of chromosomes, and was either male or female (probability 0.5). Each chromosome was 100 cM long, and had 301 marker loci, which resulted in a similar number of polymorphisms to the real dataset. A pair of parents of different sex was randomly chosen from the population to create each offspring. For each parent in a mating pair, a gamete was formed from its chromosome pairs by sampling the number of crossovers for each chromosome pair from a Poisson distribution, with mean of 1.0. Crossover points were randomly positioned along chromosome pairs. The haploid gametes were mutated at a rate of 5 × 10^-9 ^per locus per gamete per generation. If a locus was mutated, a new allele was added.

To model contemporary *B. taurus *breeds, the simulated population was subdivided in two at generation 900, both with N_e _= 200. These populations were simulated without inter mating for a further 100 generations, thus generating an inbreeding coefficient (F = 1-(1-1/2N_e_)^g^), where g = generations, relative to generation 900 of F = 0.22. In generation 1000, the difference in allele frequency was calculated for each marker. The X chromosome was not included in the simulation due to difficulties associated with the difference in N_e _for this chromosome.

#### Statistical analysis

We used the H statistic developed in Fay and Wu [14] as a frequency based test of selection from species polymorphism. The H test examines the difference between two estimates of the population genetic parameter θ, where N_e _is the effective population size (diploid) and μ is the mutation rate per generation.

(5)

The first estimate θπ is typically based on the unbiased heterozygosity in the sample [13,18]. While the second estimate θ_H _is based on the unbiased estimate of homozygosity of the derived allele in the sample [14]. However, in the case where sample numbers are sufficiently large (ie allele frequencies are based on a large number of chromosomes), it should be suitable to derive the H statistic using the uncorrected average heterozygosity (θπ) where p and 1-p = the frequency of the derived and ancestral alleles, respectively and N_L _= the number of loci.

(6)

Likewise the uncorrected average homozygosity (θ_H_) should be suitable to estimate from the sample.

(7)

Testing the frequency distribution of *B. taurus *SNP against those predicted under the neutral model was completed using a paired t-test to determine whether the mean value of θ_H _was significantly larger than θπ. Traditionally significance tests were completed using null distributions generated from computer simulations. Given the large number of loci used, the central limit theorem predicts that the test statistic will be close to a t-distribution even if the allele frequencies are not normally distributed.

Fay and Wu [14] originally modelled back mutations to account for incorrect inferences of derived and ancestral alleles. The presence of back mutations is dependent on the mutation rate and the divergence time [16]. The close relationship between *B. taurus *and the successfully amplified outgroup species should mean that double mutations were unlikely. Because the SNP assays are known to be polymorphic in *B. taurus*, the probability of a double mutation is simply the probability of a mutation at the same base in the lineage leading to Banteng, Bison and Yak. Therefore, the probability of a mutation occurring at the same site is

(8)

and the expected number of double mutations over n sites is

(9)

where u = mutation rate per year and t = generation time in years. Therefore, if u is 2.2e^-9 ^from the estimate of mammalian mutation rates by Subramanian and Kumar [36] and t is the average divergence time for Bison, Yak and Banteng, which is roughly 2.1 MY [20] then the probability for a double mutation between any of these two species and *B. taurus *is 0.005. Thus, for 7,500 bases we would expect 37 such mutations. Hence, the inferred ancestral allele should be correct over 97% of the time and are therefore assumed to be correct.

The H statistics estimated for Angus and Holstein cattle were tested for evidence of differences in the frequency spectrum of derived alleles in the two populations using a t-test. Differences in the H statistic between breeds may be due to increased selective pressure or possibly indicate differences in population substructure.

#### Frequency spectrum, the genomic distribution of derived alleles and distinguishing between the effects of positive selection, inbreeding and ascertainment bias

Fay and Wu's H statistic makes predictions regarding the frequency distribution of derived alleles under neutrality, which can be affected by population substructure, ascertainment bias or positive selection. By cataloguing the variation expected and observed with the frequency spectrum of derived alleles, inferences can be made regarding deviations from neutrality. Therefore, plots examining the spectrum of the derived polymorphism f(p) against the allele frequency (p) were compared with the theoretical value of derived polymorphisms under neutrality e(p), which is calculated as k/p and is modified from equation 4 in Fay and Wu [14], where k was chosen to match the observed values of f(p) as closely as possible. By comparing the frequency of expected and observed values of f(p), observations regarding positive selection and population substructure can be made.

One drawback of using H is that ascertainment bias can affect the frequency spectrum if there is a bias for common alleles. However, because derived and ancestral alleles are known it should be possible to examine the frequency spectrum of derived alleles devoid of any affects by ascertainment bias. The ratio f(1-p)/[f(p)+f(1-p)] should not be affected by ascertainment bias because this ratio relies on knowing the derived and ancestral allele and this information is irrelevant during SNP discovery. Therefore, f(1-p)/[f(p)+f(1-p)] was compared with the theoretical spectrum value l(p), which under neutrality was simply the proportion of derived alleles that were found to be the most common allele, or alternatively, p against p. This can be restated as: Because f(p) = k/p, f(1-p)/[f(p)+f(1-p)] = k/(1-p)/[k/p + k/(1-p)] = p.

Due to the influence of demographics on Fay and Wu's H statistic and the frequency spectrum of derived alleles, we were interested in detecting whether positive selection had driven the frequency of derived alleles in either breed of *B. taurus *at specific regions of the genome. Therefore, plots of derived allele frequency and the genomic position were examined for clustering of high frequency derived alleles. An autocorrelation of derived allele frequencies between one locus and the next on the chromosome was also completed. A positive autocorrelation should indicate that high frequency derived alleles are clustered on the genome and this may be evidence of genetic hitchhiking. To test if the observed autocorrelation could be due to inbreeding, we have examined the autocorrelation in Angus and Holstein and the simulated populations, which have been modelled to demonstrate the effect of inbreeding in the absence of selection.

#### Ancestral polymorphisms and neutral evolution

The frequency distribution of ancestral polymorphisms was examined. Ancestral polymorphisms are those that have been found to be polymorphic in Domestic cattle and also vary between the wild relatives (Bison, Yak and Banteng), and hence no ancestral allele could be determined for these sites. The frequency distribution of allele frequencies at these ancestral polymorphisms should follow a neutral model with a relatively flat distribution as they would have been segregating for ~2 million years, or since all species last shared a common ancestor. Thus the J shaped curve expected for derived mutants is not expected and a flat distribution is predicted under neutrality, as these polymorphisms are ancient with no influx of new mutations. Therefore, plots examining the frequency spectrum of ancestral alleles f(p) were compared with p and the theoretical value a(p), which was simply the mean value for f(p).

#### Differences between breeds in allele frequency

To determine if recent selection was responsible for the differences in allele frequencies between Angus and Holstein plots of Fst and average allele frequency were derived for Angus vs Holstein and the two simulated populations. The fixation index (Fst) is an estimate of population differentiation based on genetic polymorphism data. Estimates of Fst were calculated using the relationship between inbreeding and heterozygosity, which allows Fst to be calculated from genetic markers using the following equation modified from equation 13.7 in Frankham et al. [37]

(10)

where Hs is the expected heterozygosity averaged across all populations and Ht = the expected heterozygosity expected for the total population. Thus, Fst must vary from 0 to 1, which at the extremes represent fixation of alleles in different populations. If derived alleles have been fixed in one population and not in the other, as a result of positive selection, one might expect reasonably high values of Fst for alleles with extreme allele frequencies. Alternatively, if these alleles have drifted to fixation the frequency distribution of Fst should be independent of allele frequency.

## Authors' contributions

SM collected and prepared outgroup species for genotyping, analysed the SNPs and prepared the manuscript for submission. BH wrote the simulation for comparison with the real population. JM and MG coordinated the study and provided statistical and writing support. All authors read and approved the final manuscript.

## Supplementary Material

Additional file 1 **Plots for Angus and Holstein examining the frequency of the derived allele and Fst in relation to genomic position for chromosome 1.** Click here for file

Additional file 2 **Plots for Angus and Holstein examining the frequency of the derived allele and Fst in relation to genomic position for chromosome 2.** Click here for file

Additional file 3 **Plots for Angus and Holstein examining the frequency of the derived allele and Fst in relation to genomic position for chromosome 3.** Click here for file

Additional file 4 **Plots for Angus and Holstein examining the frequency of the derived allele and Fst in relation to genomic position for chromosome 4.** Click here for file

Additional file 5 **Plots for Angus and Holstein examining the frequency of the derived allele and Fst in relation to genomic position for chromosome 5.** Click here for file

Additional file 6 **Plots for Angus and Holstein examining the frequency of the derived allele and Fst in relation to genomic position for chromosome 6.** Click here for file

Additional file 7 **Plots for Angus and Holstein examining the frequency of the derived allele and Fst in relation to genomic position for chromosome 7.** Click here for file

Additional file 8 **Plots for Angus and Holstein examining the frequency of the derived allele and Fst in relation to genomic position for chromosome 8.** Click here for file

Additional file 9 **Plots for Angus and Holstein examining the frequency of the derived allele and Fst in relation to genomic position for chromosome 9.** Click here for file

Additional file 10 **Plots for Angus and Holstein examining the frequency of the derived allele and Fst in relation to genomic position for chromosome 10.** Click here for file

Additional file 11 **Plots for Angus and Holstein examining the frequency of the derived allele and Fst in relation to genomic position for chromosome 11.** Click here for file

Additional file 12 **Plots for Angus and Holstein examining the frequency of the derived allele and Fst in relation to genomic position for chromosome 12.** Click here for file

Additional file 13 **Plots for Angus and Holstein examining the frequency of the derived allele and Fst in relation to genomic position for chromosome 13.** Click here for file

Additional file 14 **Plots for Angus and Holstein examining the frequency of the derived allele and Fst in relation to genomic position for chromosome 14.** Click here for file

Additional file 15 **Plots for Angus and Holstein examining the frequency of the derived allele and Fst in relation to genomic position for chromosome 15.** Click here for file

Additional file 16 **Plots for Angus and Holstein examining the frequency of the derived allele and Fst in relation to genomic position for chromosome 16.** Click here for file

Additional file 17 **Plots for Angus and Holstein examining the frequency of the derived allele and Fst in relation to genomic position for chromosome 17.** Click here for file

Additional file 18 **Plots for Angus and Holstein examining the frequency of the derived allele and Fst in relation to genomic position for chromosome 18.** Click here for file

Additional file 19 **Plots for Angus and Holstein examining the frequency of the derived allele and Fst in relation to genomic position for chromosome 19.** Click here for file

Additional file 20 **Plots for Angus and Holstein examining the frequency of the derived allele and Fst in relation to genomic position for chromosome 20.** Click here for file

Additional file 21 **Plots for Angus and Holstein examining the frequency of the derived allele and Fst in relation to genomic position for chromosome 21.** Click here for file

Additional file 22 **Plots for Angus and Holstein examining the frequency of the derived allele and Fst in relation to genomic position for chromosome 22.** Click here for file

Additional file 23 **Plots for Angus and Holstein examining the frequency of the derived allele and Fst in relation to genomic position for chromosome 23.** Click here for file

Additional file 24 **Plots for Angus and Holstein examining the frequency of the derived allele and Fst in relation to genomic position for chromosome 24.** Click here for file

Additional file 25 **Plots for Angus and Holstein examining the frequency of the derived allele and Fst in relation to genomic position for chromosome 25.** Click here for file

Additional file 26 **Plots for Angus and Holstein examining the frequency of the derived allele and Fst in relation to genomic position for chromosome 26.** Click here for file

Additional file 27 **Plots for Angus and Holstein examining the frequency of the derived allele and Fst in relation to genomic position for chromosome 27.** Click here for file

Additional file 28 **Plots for Angus and Holstein examining the frequency of the derived allele and Fst in relation to genomic position for chromosome 28.** Click here for file

Additional file 29 **Plots for Angus and Holstein examining the frequency of the derived allele and Fst in relation to genomic position for chromosome 29.** Click here for file

Additional file 30 **Plots for Angus and Holstein examining the frequency of the derived allele and Fst in relation to genomic position for chromosome X.** Click here for file
