# Supplementary figures and images for: An examination of positive selection and changing effective population size in Angus and Holstein cattle populations (Bos taurus) using a high density SNP genotyping platform and the contribution of ancient polymorphism to genomic diversity in Domestic cattle
Source: BMC Genomics. 2009 Apr 24;10:181. doi: 10.1186/1471-2164-10-181 (PMC2681480; doi:10.1186/1471-2164-10-181)

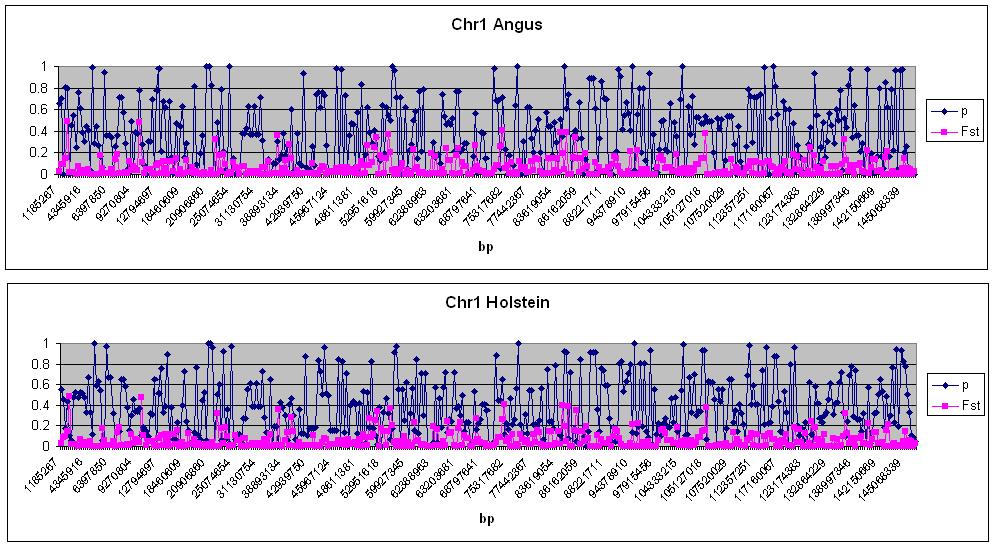

Supplement: Additional file 1 — Plots for Angus and Holstein examining the frequency of the derived allele and Fst in relation to genomic position for chromosome 1. [file 1471-2164-10-181-S1.jpeg]

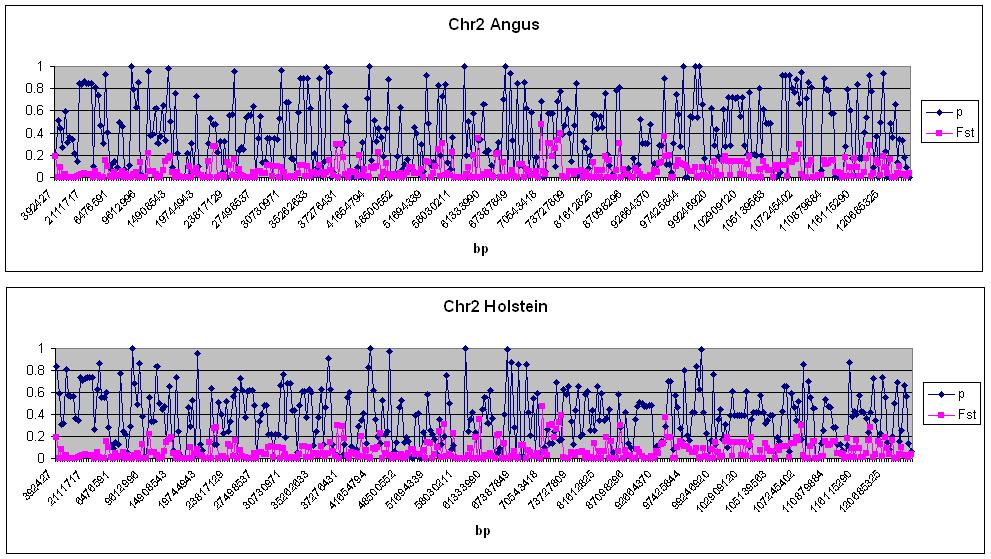

Supplement: Additional file 2 — Plots for Angus and Holstein examining the frequency of the derived allele and Fst in relation to genomic position for chromosome 2. [file 1471-2164-10-181-S2.jpeg]

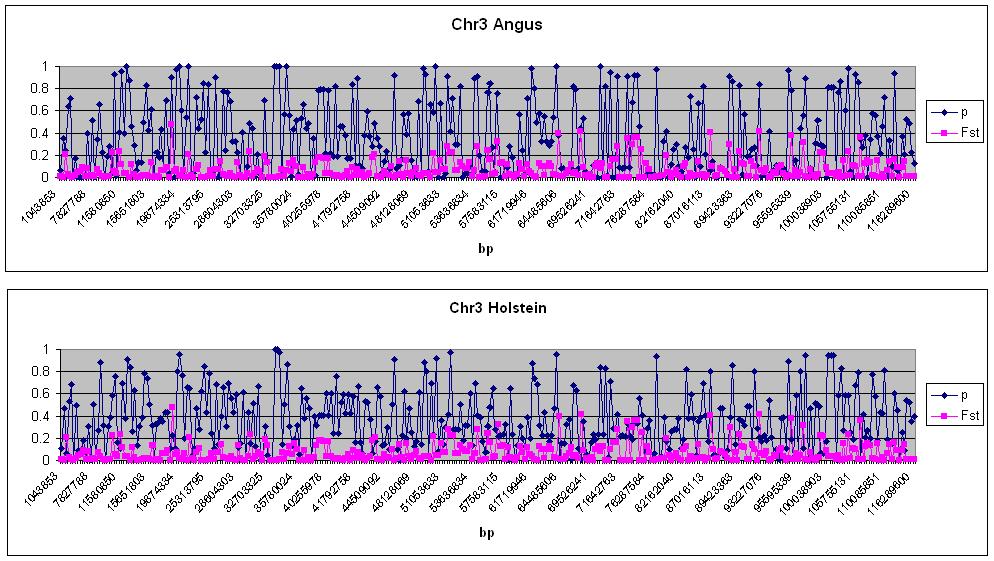

Supplement: Additional file 3 — Plots for Angus and Holstein examining the frequency of the derived allele and Fst in relation to genomic position for chromosome 3. [file 1471-2164-10-181-S3.jpeg]

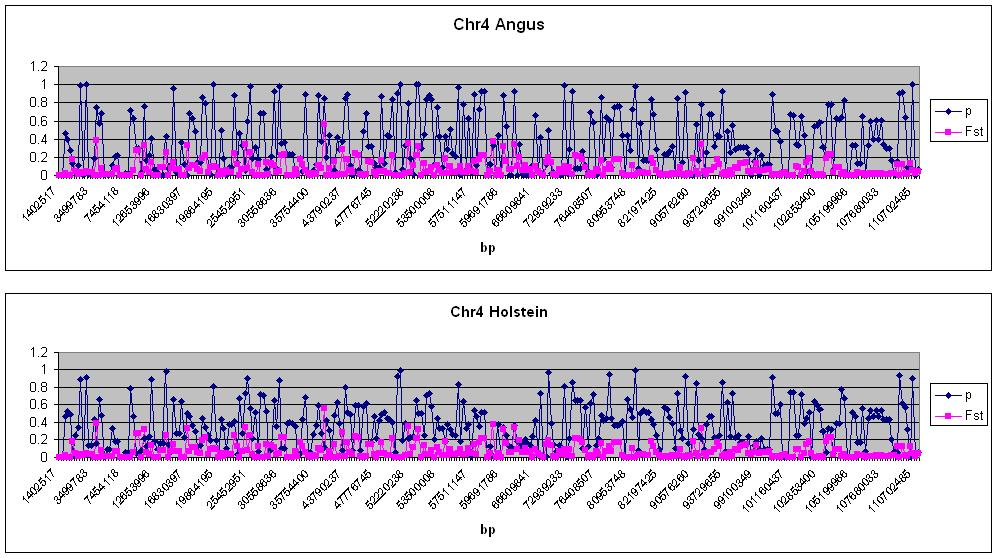

Supplement: Additional file 4 — Plots for Angus and Holstein examining the frequency of the derived allele and Fst in relation to genomic position for chromosome 4. [file 1471-2164-10-181-S4.jpeg]

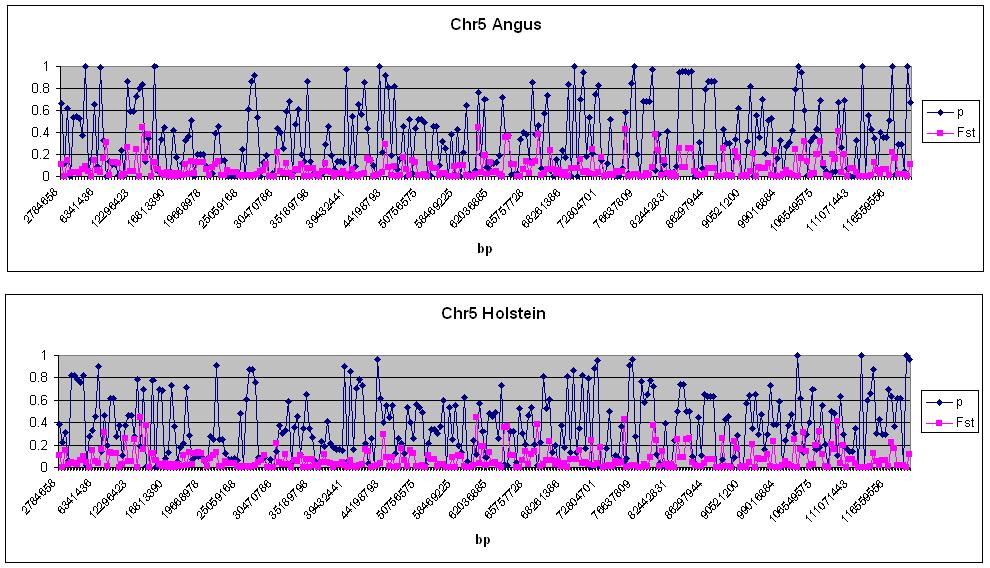

Supplement: Additional file 5 — Plots for Angus and Holstein examining the frequency of the derived allele and Fst in relation to genomic position for chromosome 5. [file 1471-2164-10-181-S5.jpeg]

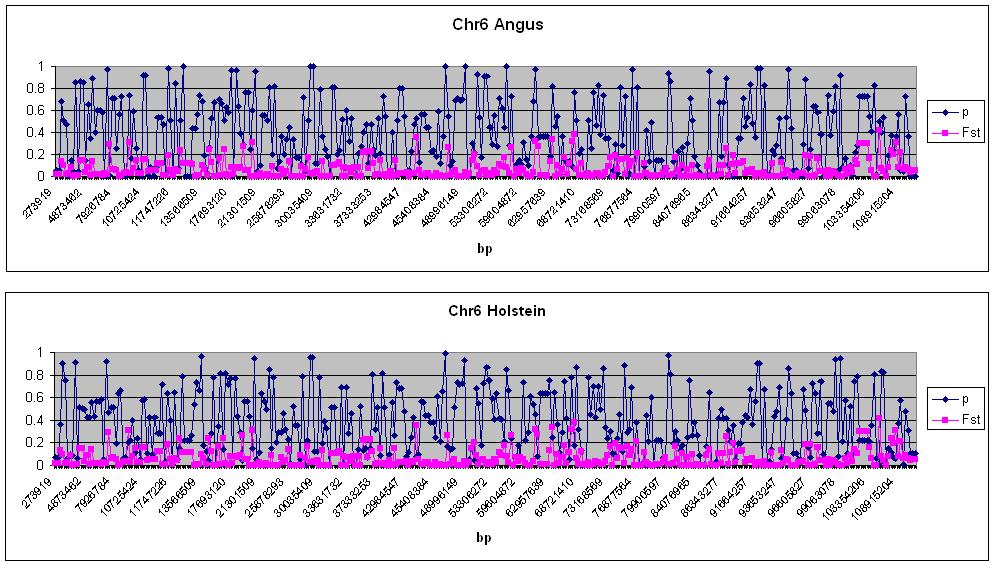

Supplement: Additional file 6 — Plots for Angus and Holstein examining the frequency of the derived allele and Fst in relation to genomic position for chromosome 6. [file 1471-2164-10-181-S6.jpeg]

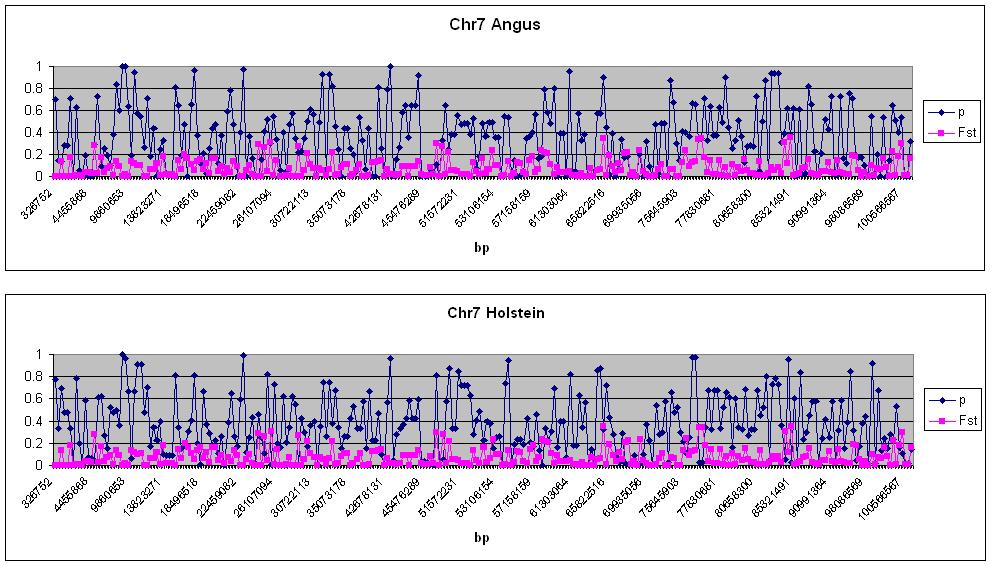

Supplement: Additional file 7 — Plots for Angus and Holstein examining the frequency of the derived allele and Fst in relation to genomic position for chromosome 7. [file 1471-2164-10-181-S7.jpeg]

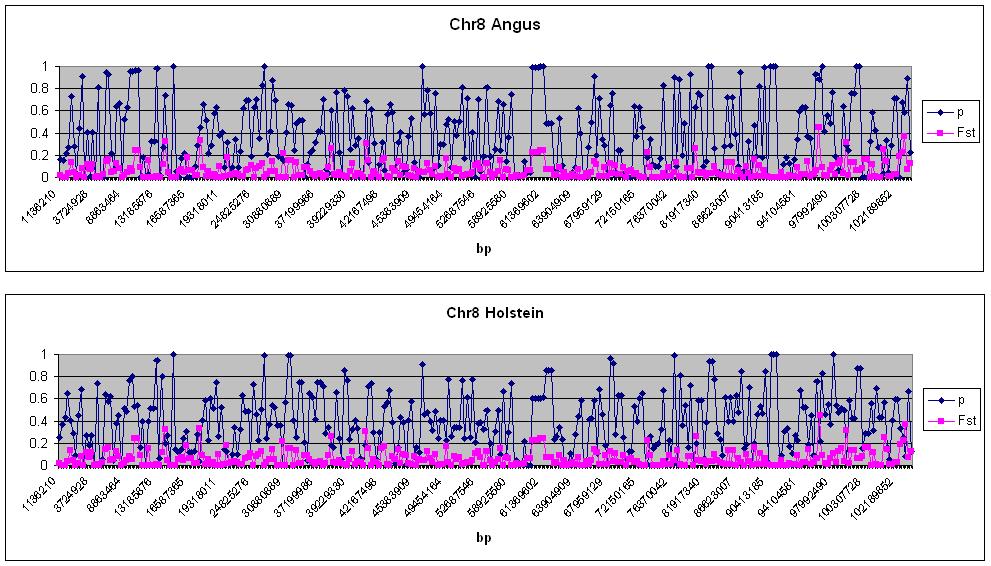

Supplement: Additional file 8 — Plots for Angus and Holstein examining the frequency of the derived allele and Fst in relation to genomic position for chromosome 8. [file 1471-2164-10-181-S8.jpeg]

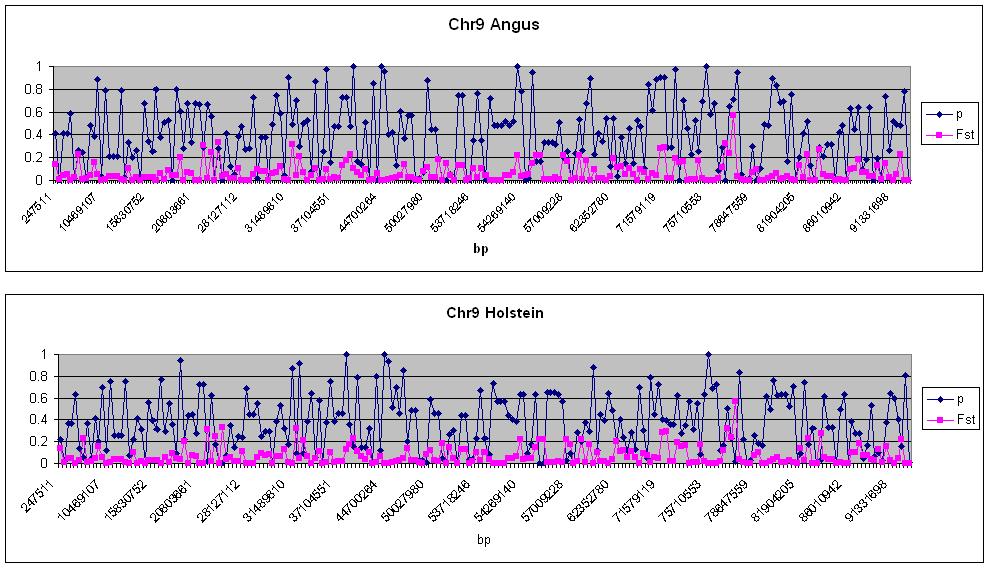

Supplement: Additional file 9 — Plots for Angus and Holstein examining the frequency of the derived allele and Fst in relation to genomic position for chromosome 9. [file 1471-2164-10-181-S9.jpeg]

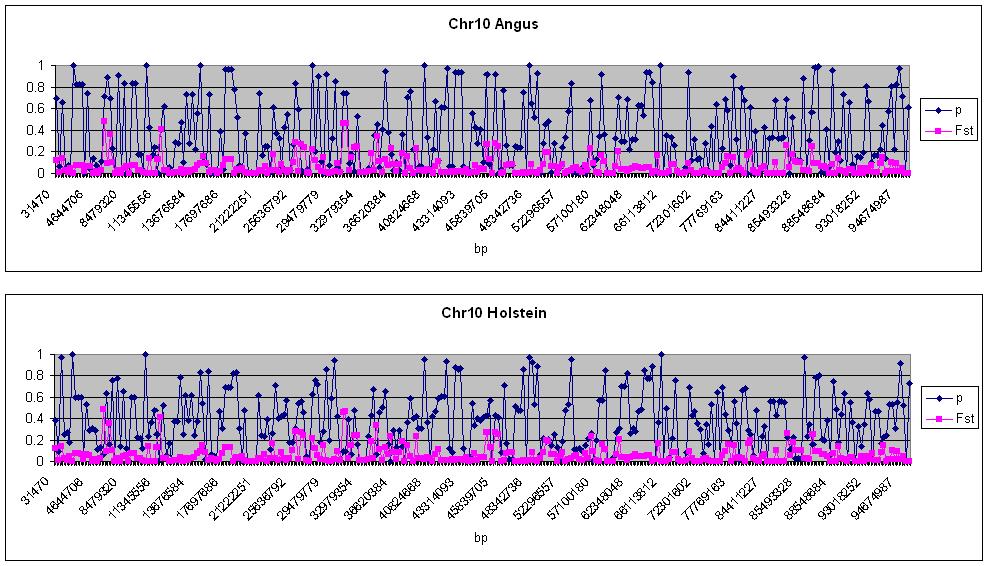

Supplement: Additional file 10 — Plots for Angus and Holstein examining the frequency of the derived allele and Fst in relation to genomic position for chromosome 10. [file 1471-2164-10-181-S10.jpeg]

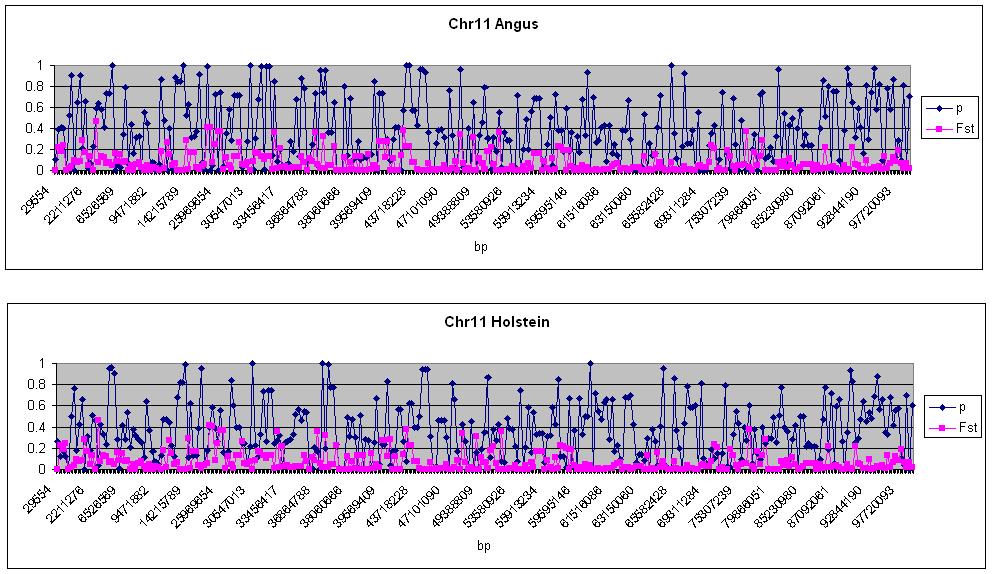

Supplement: Additional file 11 — Plots for Angus and Holstein examining the frequency of the derived allele and Fst in relation to genomic position for chromosome 11. [file 1471-2164-10-181-S11.jpeg]

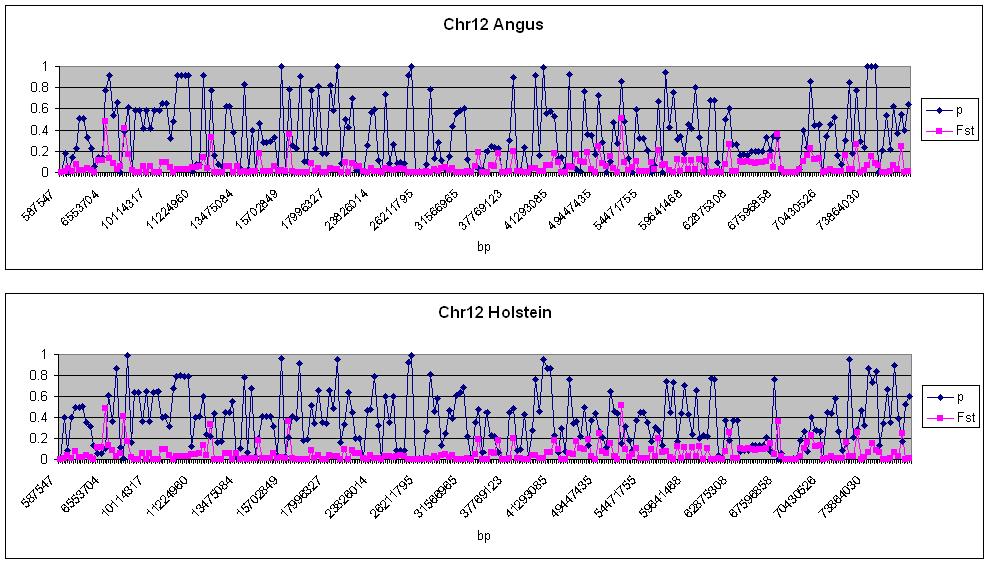

Supplement: Additional file 12 — Plots for Angus and Holstein examining the frequency of the derived allele and Fst in relation to genomic position for chromosome 12. [file 1471-2164-10-181-S12.jpeg]

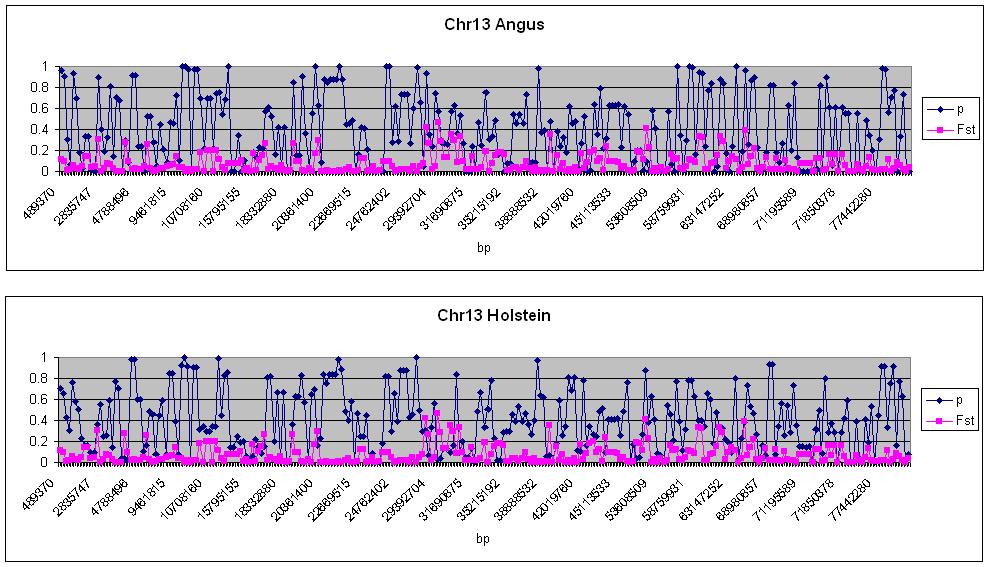

Supplement: Additional file 13 — Plots for Angus and Holstein examining the frequency of the derived allele and Fst in relation to genomic position for chromosome 13. [file 1471-2164-10-181-S13.jpeg]

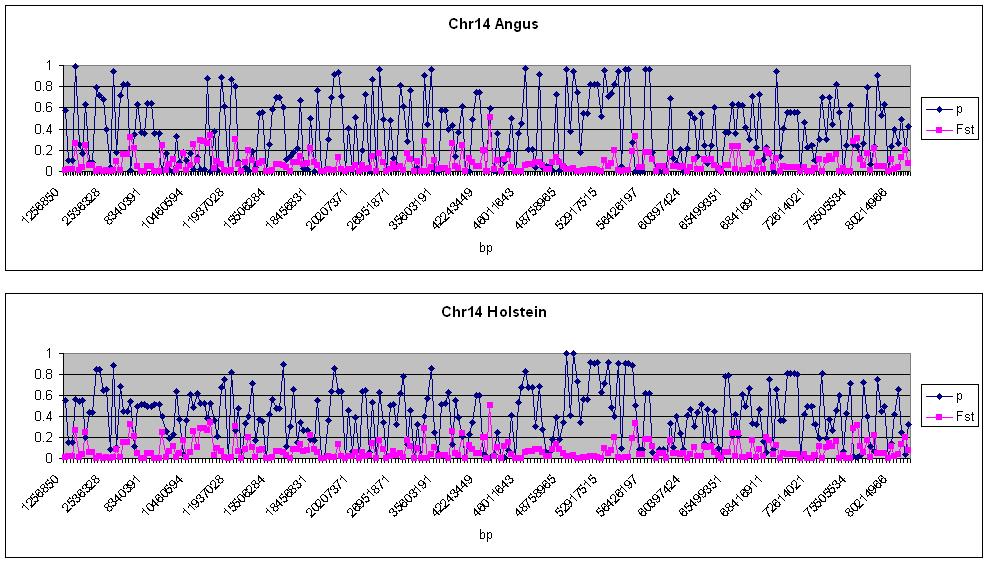

Supplement: Additional file 14 — Plots for Angus and Holstein examining the frequency of the derived allele and Fst in relation to genomic position for chromosome 14. [file 1471-2164-10-181-S14.jpeg]

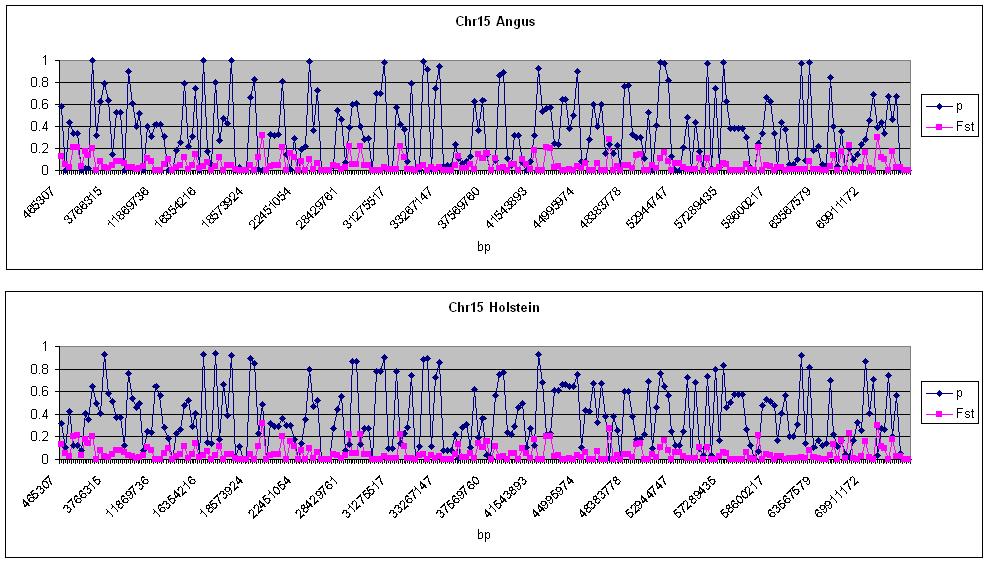

Supplement: Additional file 15 — Plots for Angus and Holstein examining the frequency of the derived allele and Fst in relation to genomic position for chromosome 15. [file 1471-2164-10-181-S15.jpeg]

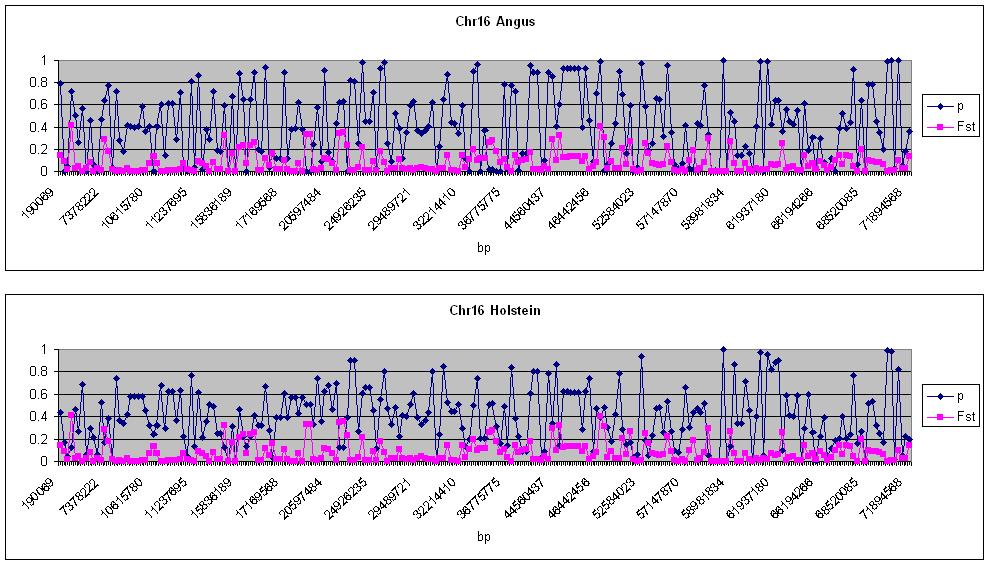

Supplement: Additional file 16 — Plots for Angus and Holstein examining the frequency of the derived allele and Fst in relation to genomic position for chromosome 16. [file 1471-2164-10-181-S16.jpeg]

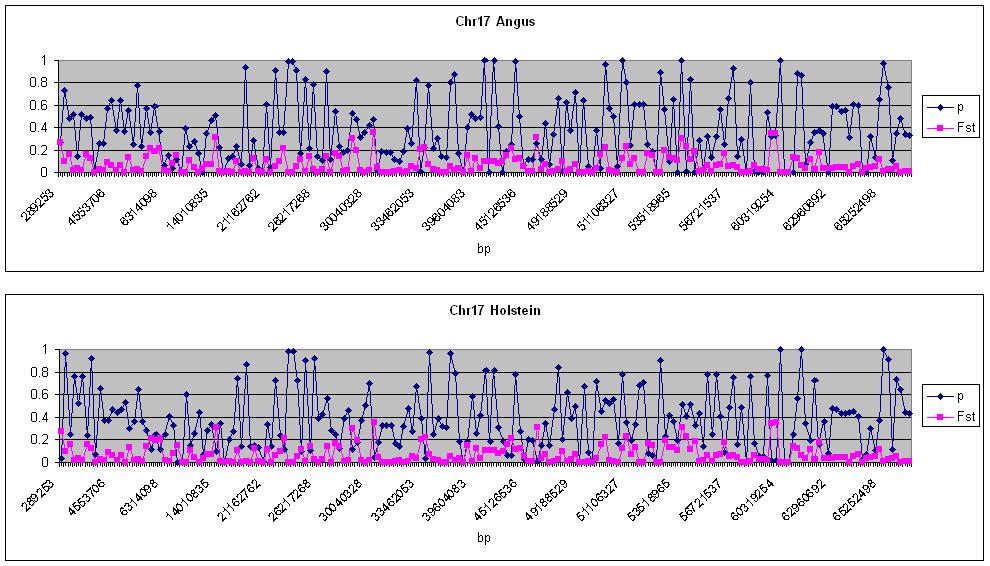

Supplement: Additional file 17 — Plots for Angus and Holstein examining the frequency of the derived allele and Fst in relation to genomic position for chromosome 17. [file 1471-2164-10-181-S17.jpeg]

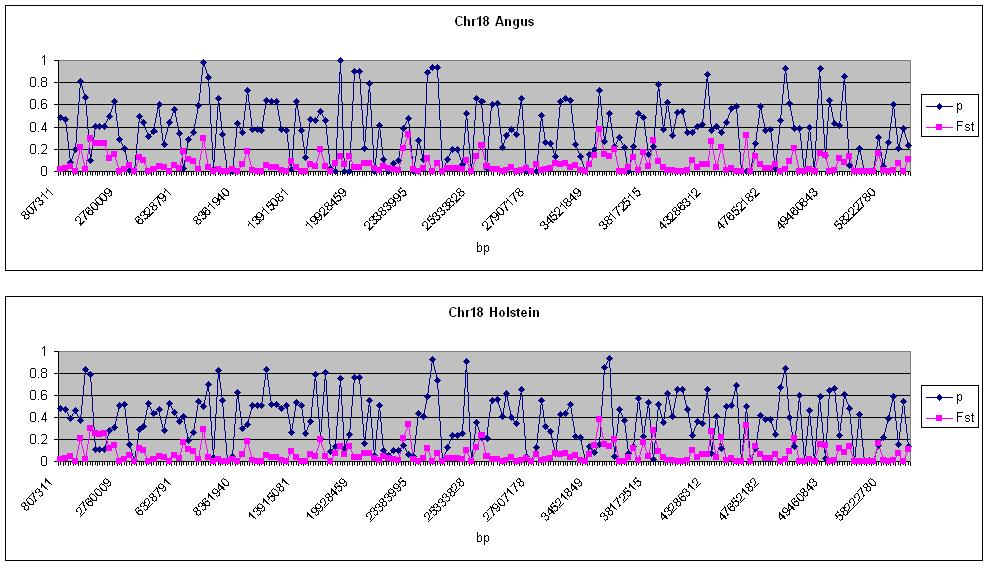

Supplement: Additional file 18 — Plots for Angus and Holstein examining the frequency of the derived allele and Fst in relation to genomic position for chromosome 18. [file 1471-2164-10-181-S18.jpeg]

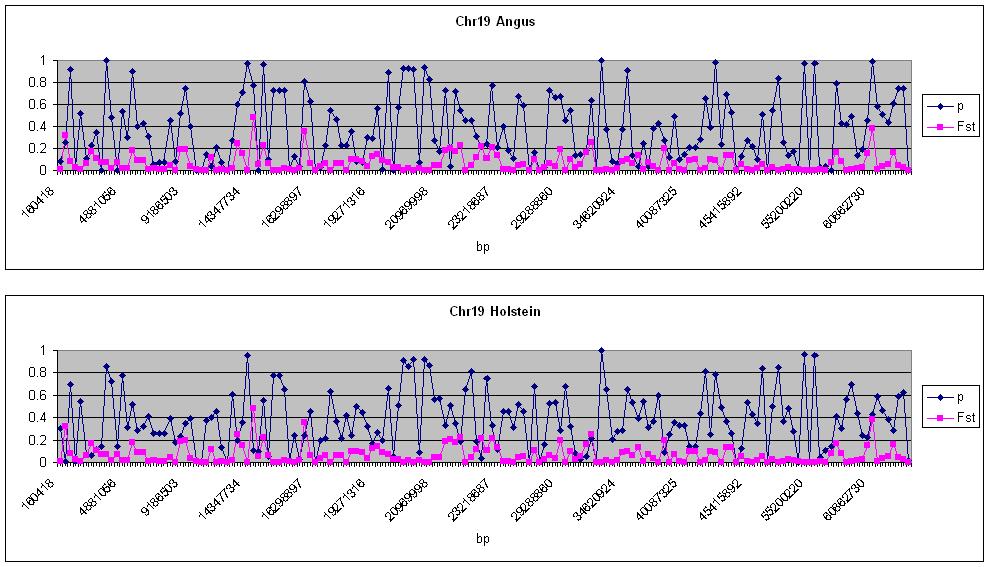

Supplement: Additional file 19 — Plots for Angus and Holstein examining the frequency of the derived allele and Fst in relation to genomic position for chromosome 19. [file 1471-2164-10-181-S19.jpeg]

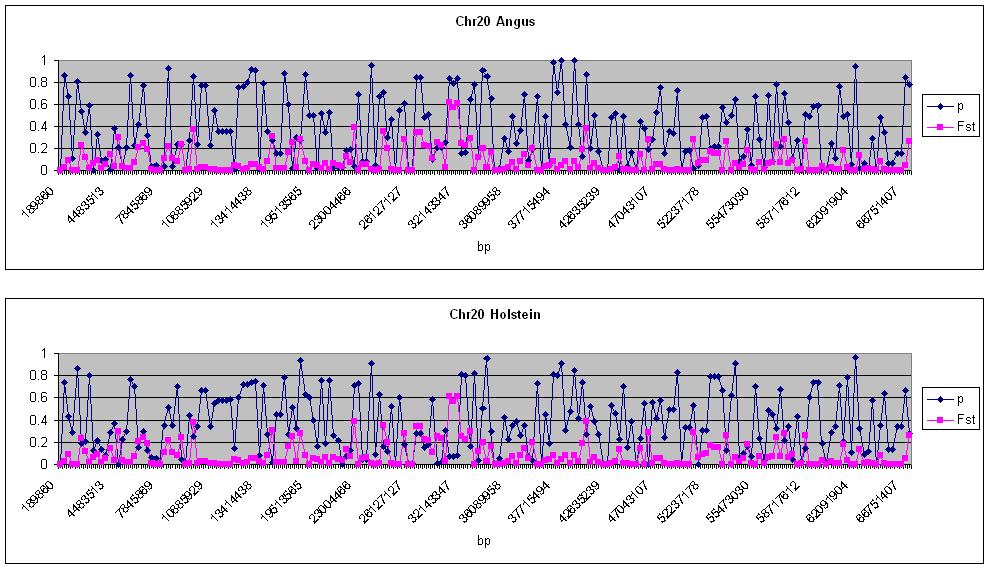

Supplement: Additional file 20 — Plots for Angus and Holstein examining the frequency of the derived allele and Fst in relation to genomic position for chromosome 20. [file 1471-2164-10-181-S20.jpeg]

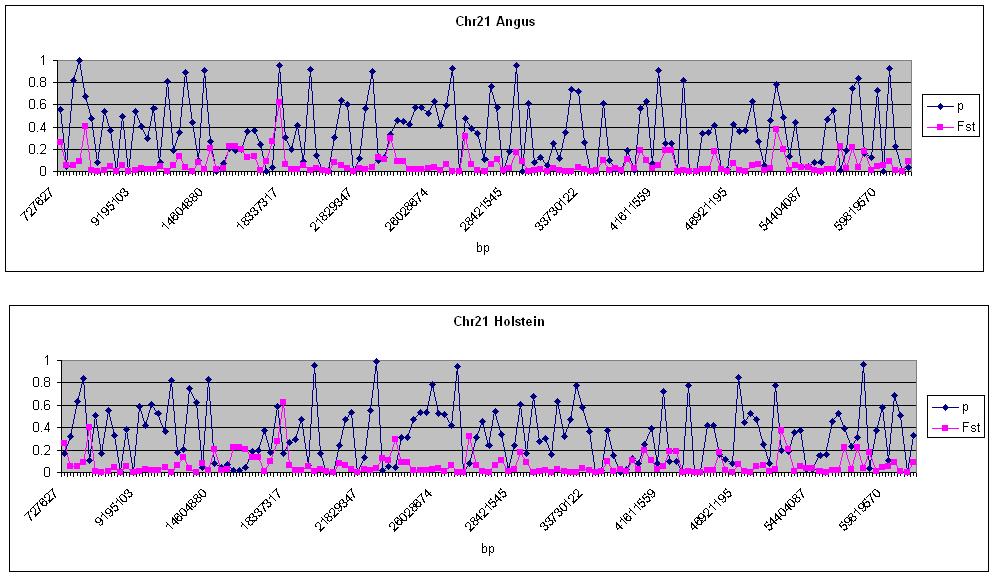

Supplement: Additional file 21 — Plots for Angus and Holstein examining the frequency of the derived allele and Fst in relation to genomic position for chromosome 21. [file 1471-2164-10-181-S21.jpeg]

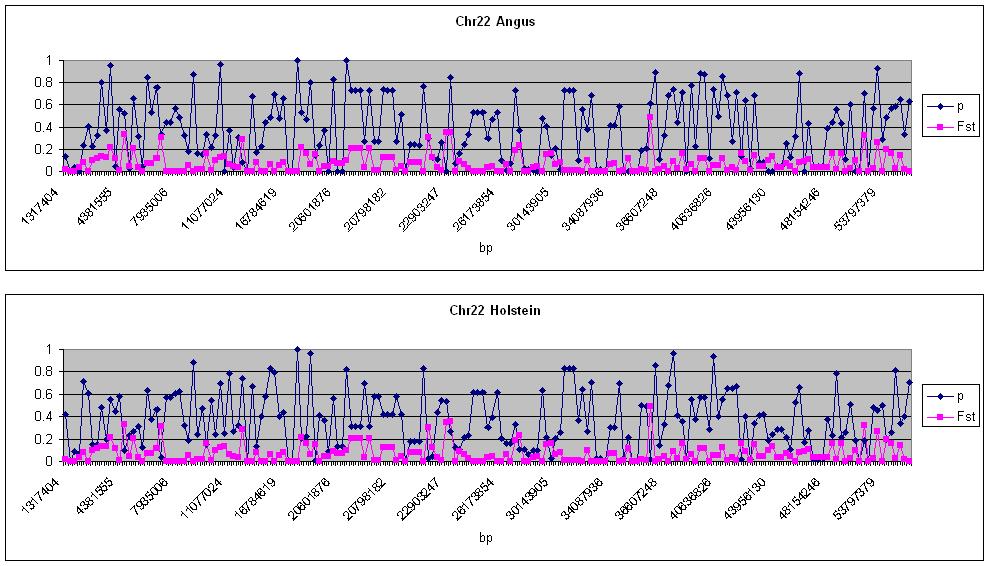

Supplement: Additional file 22 — Plots for Angus and Holstein examining the frequency of the derived allele and Fst in relation to genomic position for chromosome 22. [file 1471-2164-10-181-S22.jpeg]

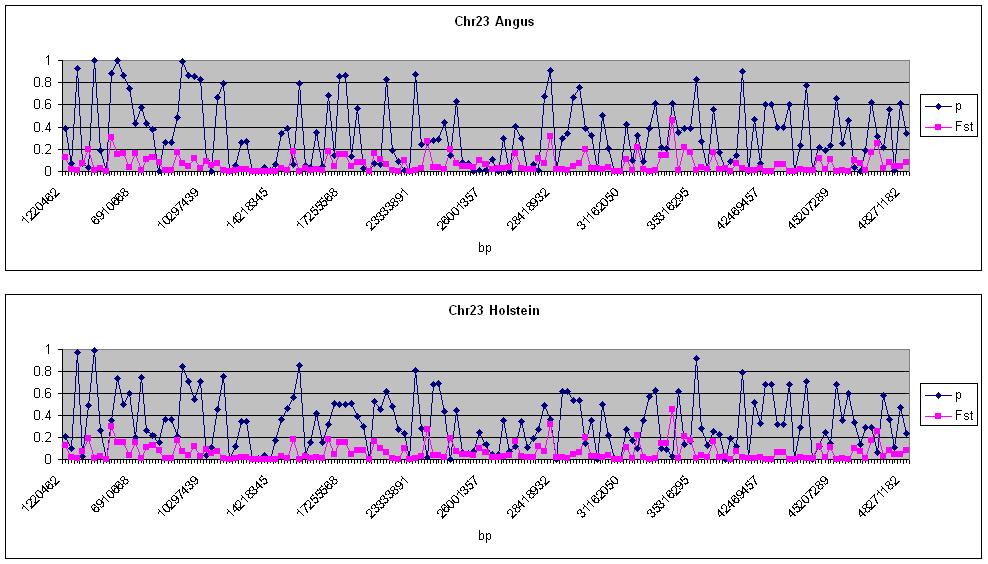

Supplement: Additional file 23 — Plots for Angus and Holstein examining the frequency of the derived allele and Fst in relation to genomic position for chromosome 23. [file 1471-2164-10-181-S23.jpeg]

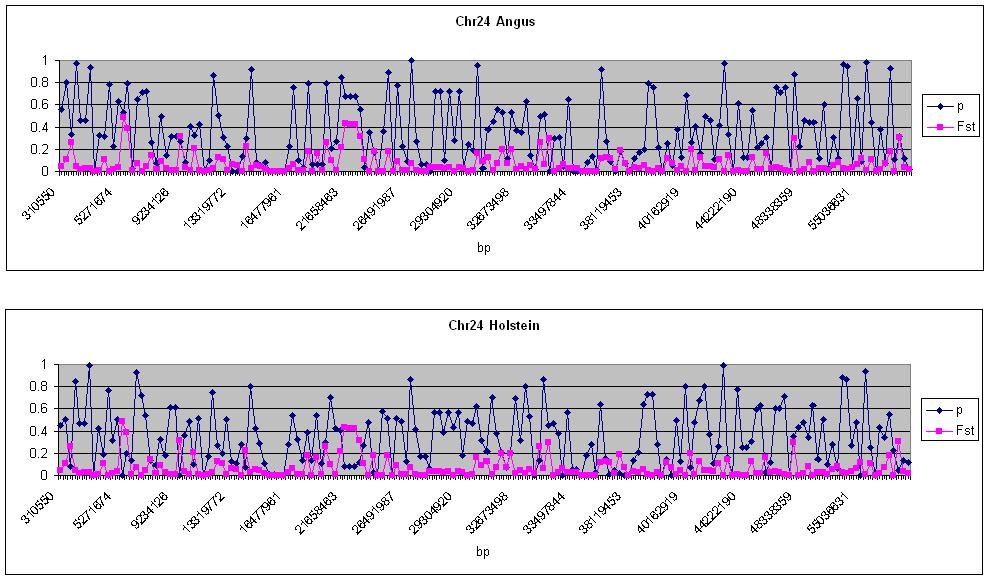

Supplement: Additional file 24 — Plots for Angus and Holstein examining the frequency of the derived allele and Fst in relation to genomic position for chromosome 24. [file 1471-2164-10-181-S24.jpeg]

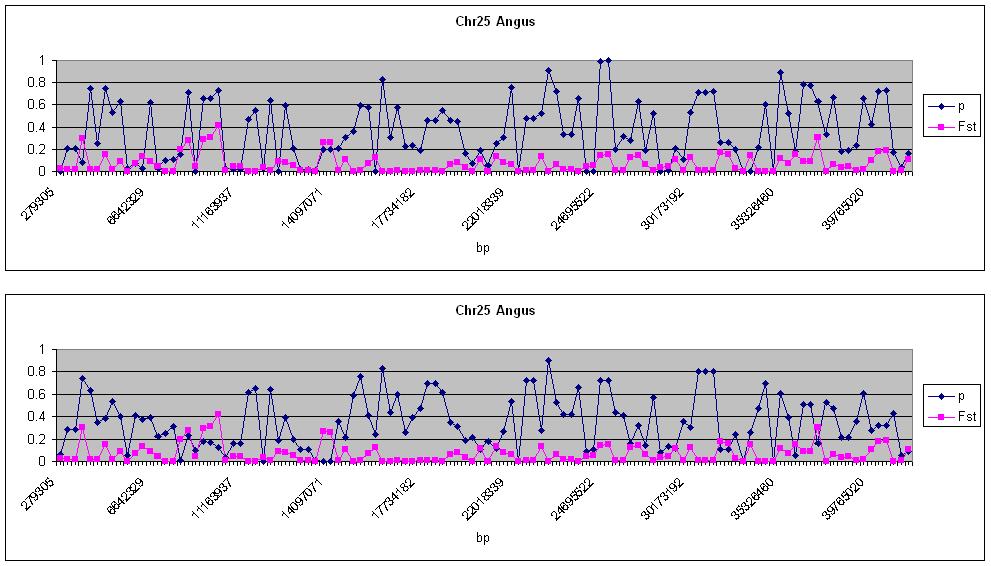

Supplement: Additional file 25 — Plots for Angus and Holstein examining the frequency of the derived allele and Fst in relation to genomic position for chromosome 25. [file 1471-2164-10-181-S25.jpeg]

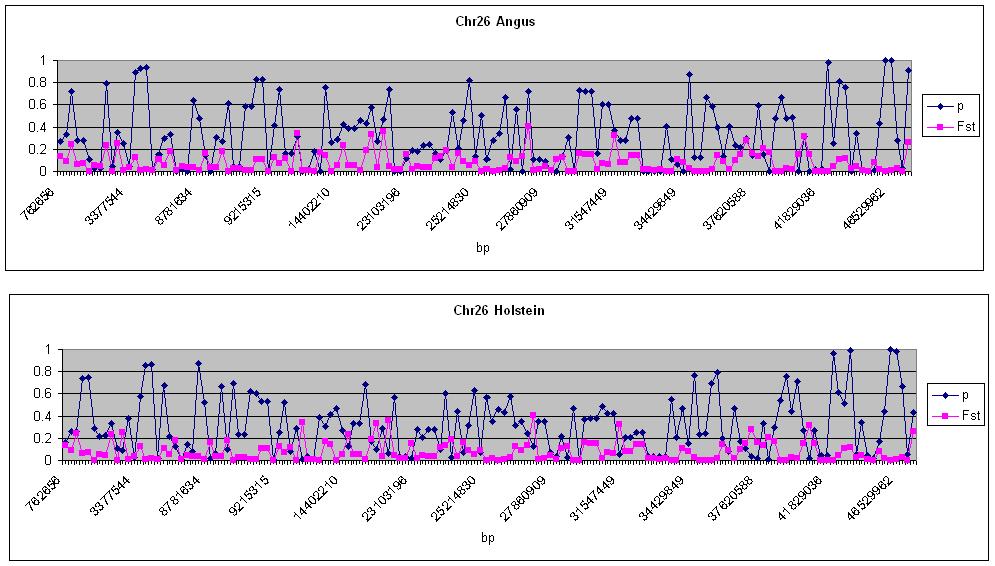

Supplement: Additional file 26 — Plots for Angus and Holstein examining the frequency of the derived allele and Fst in relation to genomic position for chromosome 26. [file 1471-2164-10-181-S26.jpeg]

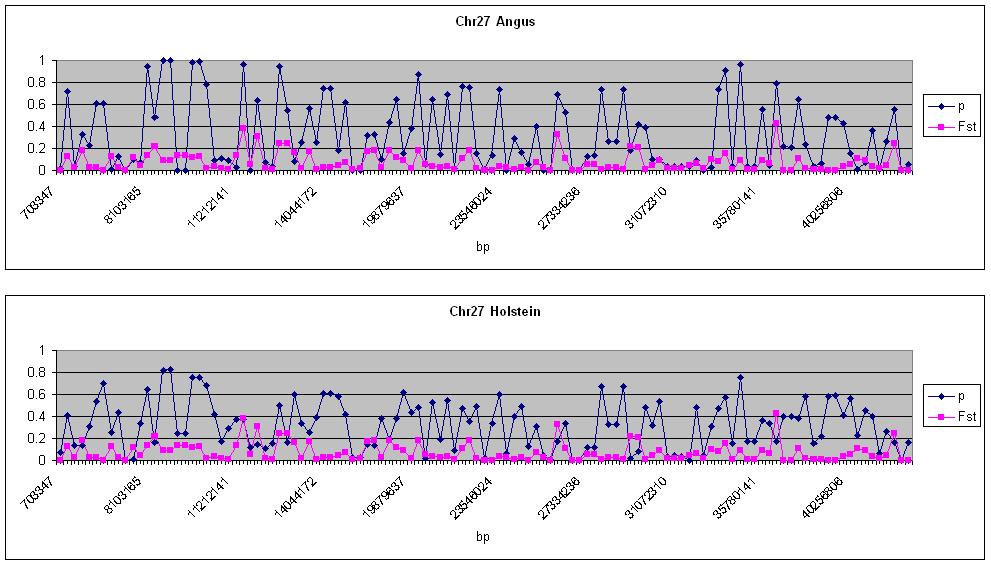

Supplement: Additional file 27 — Plots for Angus and Holstein examining the frequency of the derived allele and Fst in relation to genomic position for chromosome 27. [file 1471-2164-10-181-S27.jpeg]

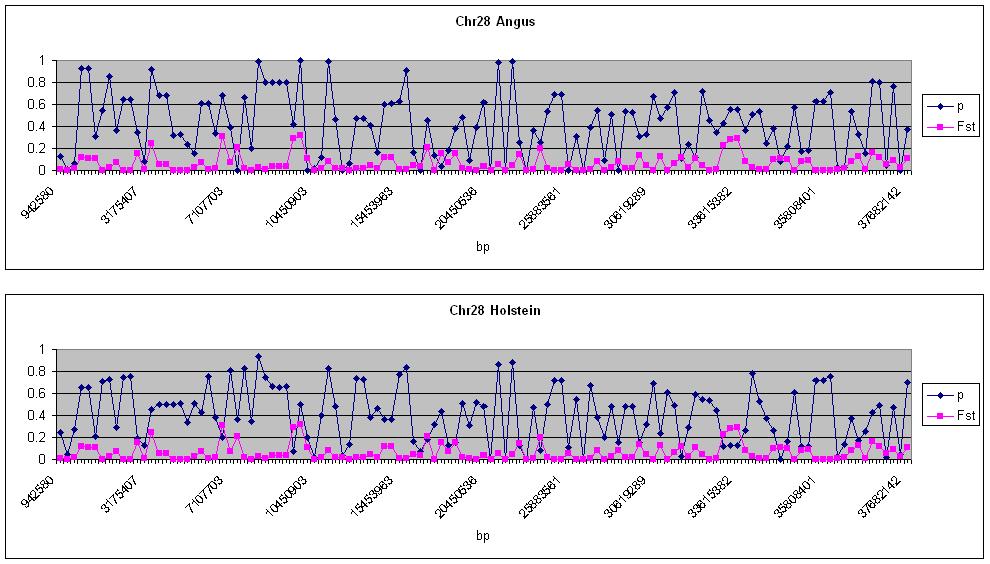

Supplement: Additional file 28 — Plots for Angus and Holstein examining the frequency of the derived allele and Fst in relation to genomic position for chromosome 28. [file 1471-2164-10-181-S28.jpeg]

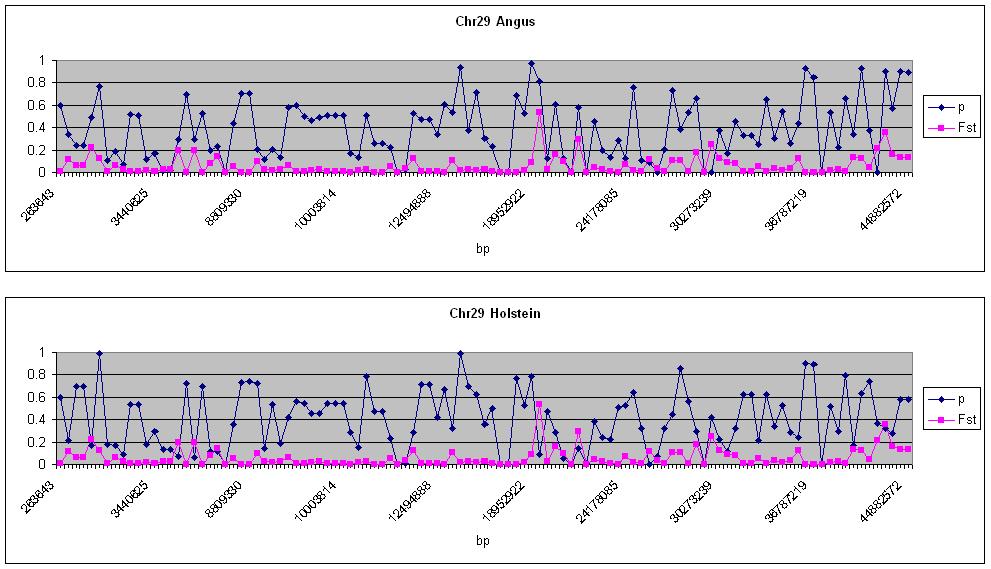

Supplement: Additional file 29 — Plots for Angus and Holstein examining the frequency of the derived allele and Fst in relation to genomic position for chromosome 29. [file 1471-2164-10-181-S29.jpeg]

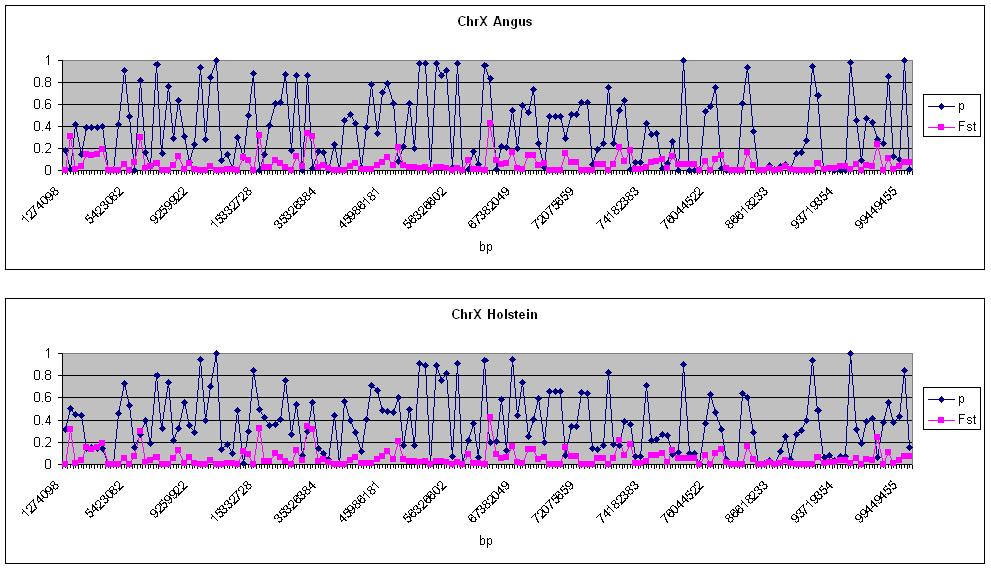

Supplement: Additional file 30 — Plots for Angus and Holstein examining the frequency of the derived allele and Fst in relation to genomic position for chromosome X. [file 1471-2164-10-181-S30.jpeg]
